# Simultaneous Quantitative MRI Mapping of *T*_1_, *T*_2_* and Magnetic Susceptibility with Multi-Echo MP2RAGE

**DOI:** 10.1371/journal.pone.0169265

**Published:** 2017-01-12

**Authors:** Riccardo Metere, Tobias Kober, Harald E. Möller, Andreas Schäfer

**Affiliations:** 1 Max Planck Institute for Human Cognitive and Brain Sciences, Leipzig, Germany; 2 Advanced Clinical Imaging Technology, Siemens Healthcare HC CMEA SUI DI BM PI, Lausanne, Switzerland; 3 Department of Radiology, University Hospital (CHUV), Lausanne, Switzerland; 4 LTS5, École Polytechnique Fédérale de Lausanne, Lausanne, Switzerland; 5 Diagnostic Imaging—Magnetic Resonance—Research & Development, Siemens Healthcare GmbH, Erlangen, Germany; Mathematical Institute, HUNGARY

## Abstract

The knowledge of relaxation times is essential for understanding the biophysical mechanisms underlying contrast in magnetic resonance imaging. Quantitative experiments, while offering major advantages in terms of reproducibility, may benefit from simultaneous acquisitions. In this work, we demonstrate the possibility of simultaneously recording relaxation-time and susceptibility maps with a prototype Multi-Echo (ME) Magnetization-Prepared 2 RApid Gradient Echoes (MP2RAGE) sequence. *T*_1_ maps can be obtained using the MP2RAGE sequence, which is relatively insensitive to inhomogeneities of the radio-frequency transmit field, $B_{1}^{+}$. As an extension, multiple gradient echoes can be acquired in each of the MP2RAGE readout blocks, which permits the calculation of $T_{2}^{*}$ and susceptibility maps. We used computer simulations to explore the effects of the parameters on the precision and accuracy of the mapping. *In vivo* parameter maps up to 0.6 mm nominal resolution were acquired at 7 T in 19 healthy volunteers. Voxel-by-voxel correlations and the test-retest reproducibility were used to assess the reliability of the results. When using optimized paramenters, *T*_1_ maps obtained with ME-MP2RAGE and standard MP2RAGE showed excellent agreement for the whole range of values found in brain tissues. Simultaneously obtained $T_{2}^{*}$ and susceptibility maps were of comparable quality as Fast Low-Angle SHot (FLASH) results. The acquisition times were more favorable for the ME-MP2RAGE (≈ 19 min) sequence as opposed to the sum of MP2RAGE (≈ 12 min) and FLASH (≈ 10 min) acquisitions. Without relevant sacrifice in accuracy, precision or flexibility, the multi-echo version may yield advantages in terms of reduced acquisition time and intrinsic co-registration, provided that an appropriate optimization of the acquisition parameters is performed.

## 1 Introduction

Quantitative mapping of Magnetic Resonance (MR) relaxation times has become a useful tool in brain research as well as for clinical applications due to the possibility of directly comparing results across subjects and sites. The knowledge of relaxation times is further essential for understanding the biophysical mechanisms underlying image contrast. Recently, the correlation between relaxation times and brain tissue composition has been highlighted [1, 2]. For example, it has been shown that the effective transverse relaxation time, $T_{2}^{*}$, is influenced by iron and myelin content, whereas the longitudinal relaxation time, *T*_1_, depends on myelin but only little on iron content [2, 3]. Three-Dimensional (3D) *T*_1_ maps are also frequently used for differentiating brain tissue types, especially for White Matter (WM), Gray Matter (GM), and Cerebro-Spinal Fluid (CSF) segmentation [3–6].

*T*_1_ maps can be obtained, for example, with ‘Fast Low-Angle SHot’ (FLASH) [7, 8] employing different flip angles [9] or with inversion-recovery sequences [10]. If the recovery curve is sampled with sufficient density, separation of multiple water compartments in the brain based on their *T*_1_ values has been achieved by relaxographic techniques [11, 12]. Recently, the ‘Magnetization-Prepared 2 RApid Gradient Echoes’ (MP2RAGE) sequence has been proposed for an efficient 3D mapping of *T*_1_ [5, 13]. It consists of two Gradient-Recalled Echo (GRE) image volumes acquired within a single repetition at two different inversion times, *T*_*I*,1_ and *T*_*I*,2_. Assuming that the recovery can be characterized by a single exponential, accurate *T*_1_ estimates are obtained as long as the acquisition parameters are properly chosen, allowing for sufficient insensitivity to inhomogeneities of the Radio-Frequency (RF) transmit magnetic field amplitude, $B_{1}^{+}$.

Mapping of $T_{2}^{*}$ requires a gradient acquisition scheme with multiple echoes sampled at different times, *T*_*E*,*i*_. Usually, the FLASH sequence with multi-echo (ME) readout is employed for $T_{2}^{*}$-related relaxometry, because of its relatively high robustness against inhomogeneities of the main magnetic field amplitude, *B*_0_.

In recent years, Quantitative Susceptibility Mapping (QSM) has been developed to measure another intrinsic property of tissues, the bulk magnetic susceptibility, *χ* [14–19]. It employs maps of the spatial variation of *B*_0_ extracted from the signal phase of a GRE [20]. Molecular anisotropy of the susceptibility and orientation of micro-structural components have been shown to influence the phase contrast as well [18, 21–27]. Furthermore, QSM has led to the possibility of differentiating between diamagnetic myelin and paramagnetic iron deposition [17, 28–31] that is already exploited in brain research and clinical applications.

As a typical drawback, many techniques for quantitative mapping of tissue parameters require longer scan times than conventional *T*_1_-, $T_{2}^{*}$- or susceptibility-weighted MR Imaging (MRI). This imposes limitations in clinical studies where the available scan time is often restricted. Hence, more versatile sequence implementations permitting the simultaneous acquisition of image data with different contrasts are of interest. Previous work has shown that collecting multiple echoes in conventional ‘Magnetization-Prepared RApid Gradient Echo’ (MP-RAGE) imaging [32] may be helpful in brain morphometry studies [33, 34]. In this work, we introduce an ME version of the MP2RAGE sequence that permits simultaneous 3D mapping of *T*_1_, $T_{2}^{*}$, and *χ*. Computer simulations and experimental verification in healthy human brains at 7T demonstrate that all parameters can be obtained with similar accuracy as achieved using separately acquired MP2RAGE and ME-FLASH scans.

## 2 Methods

In order to investigate whether the ME-MP2RAGE sequence permits robust mapping of *T*_1_, $T_{2}^{*}$ and *χ*, we used a two–step approach: *(i)* the effects of the acquisition parameters on the accuracy and precision of the maps were investigated by simulations and experiments, and *(ii)* after finding a suitable set of parameters, the reproducibility of the maps was evaluated in comparison to standard MP2RAGE or ME-FLASH results.

### 2.1 Description of the Pulse Sequence

**Fig 1:**
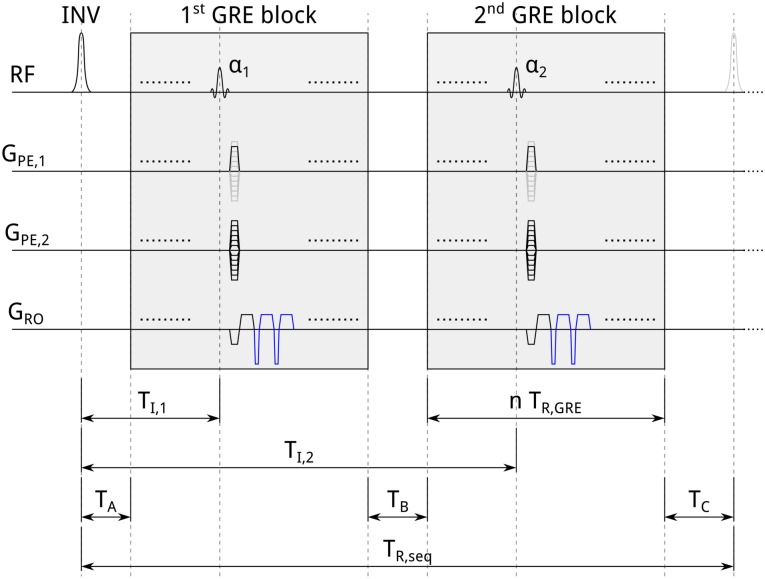
Schematic diagram of the ME-MP2RAGE sequence. After an adiabatic inversion pulse (INV), two GRE readout blocks are collected with excitation pulse flip angles, *α*_1_ and *α*_2_. Both GRE blocks consist of *n* acquisitions of *k*-space lines, each of duration *T*_*R*,*GRE*_, stepping linearly through the second phase-encoding direction (as in standard MP2RAGE). To each *T*_*R*,*seq*_ corresponds a *k*-space line acquisition for the first phase-encoding direction. The center of *k*-space is acquired at times *T*_*I*,1_ and *T*_*I*,2_. Examples of the additional mono-polar gradient lobes of the ME readouts are indicated by blue color. The sequence repetition time, *T*_*R*,*seq*_, is defined as the time between two successive inversion pulses. The total acquisition time is thus defined by *T*_*R*,*seq*_ multiplied by the number of steps in the second phase-encoding direction. *T*_*A*_, *T*_*B*_, and *T*_*C*_ denote, respectively, the durations from the initial inversion pulse to the onset of the first GRE block, between the two GRE blocks, and from the end of the second GRE block to the next inversion pulse. Note that the first and the second phase-encoding direction may be interchanged.

The ME-MP2RAGE pulse sequence, shown in Fig 1, is obtained from MP2RAGE by acquiring multiple gradient echoes for each phase-encoding step. The signal expression for MP2RAGE derived in [5] is also valid for ME-MP2RAGE. Briefly, the signals (at any echo time, *T*_*E*,*i*_) can be written as:

$$\begin{array}{ll} S\left(T_{I,1}\right) & =\xi M_{0}\text{exp}\left(-T_{E,i}/T_{2}^{*}\right)\text{sin }\alpha_{1}\\ & \cdot \{\left[\frac{-\eta M_{z}^{ss}}{M_{0}}E_{A}+\left(1-E_{A}\right)\right]{\left(E_{1}\text{cos }\alpha_{1}\right)}^{\frac{n}{2}-1}\\ & +\left(1-E_{1}\right)\frac{1-{\left(E_{1}\text{cos }\alpha_{1}\right)}^{\frac{n}{2}-1}}{1-E_{1}\text{cos }\alpha_{1}}\} \end{array}$$ (1)

and

$$\begin{array}{ll} S\left(T_{I,2}\right) & =\xi M_{0}\text{ exp}\left(-T_{E,i}/T_{2}^{*}\right)\text{sin }\alpha_{2}\\ & \cdot \{\left[\frac{M_{z}^{ss}}{M_{0}}E_{C}^{-1}+\left(1-E_{C}^{-1}\right)\right]{\left(E_{1}\text{cos }\alpha_{2}\right)}^{-\frac{n}{2}}\\ & +\left(1-E_{1}\right)\frac{1-{\left(E_{1}\text{cos }\alpha_{2}\right)}^{-\frac{n}{2}}}{1-E_{1}\text{cos }\alpha_{2}}\}, \end{array}$$ (2)

where *M*_0_ is the equilibrium magnetization, *E*_1_ ≡ exp(−*T*_*R*,*GRE*_/*T*_1_), *E*_*A*_ ≡ exp(−*T*_*A*_/*T*_1_), *E*_*C*_ ≡ exp(−*T*_*C*_/*T*_1_), and *n* is the number of *k*-space lines in one GRE block. The intervals *T*_*R*,*GRE*_, *T*_*A*_, and *T*_*C*_, as well as the flip angles *α*_1_ and *α*_2_ are defined in Fig 1; *η* ≡ (1/2)[1 − *M*_*z*_(0^+^)/*M*_*z*_(0^−^)] is the inversion efficiency of the adiabatic pulse, where *M*_*z*_(0^−^) and *M*_*z*_(0^+^) are the longitudinal magnetizations immediately before and after the application of the pulse, respectively [35]. $M_{z}^{ss}$ is the steady-state longitudinal magnetization, which is obtained by solving the Bloch equations with appropriate boundary conditions (see Eq A1.4 in [5]). Finally, all scanner-dependent parameters required for converting magnetization into signal voltage are lumped together in the scaling constant *ξ*.

The (complex) signals from the two inversion-contrast image volumes are combined by computing

ρ=Re|S*(TI,1)·S(TI,2)|S(TI,1)|2+|S(TI,2)|2, (3)

where S* denotes the complex conjugate of *S* and Re[x] returns the real part of *x*. The expression for *ρ* can be found analytically, and it is used to invert the equation for *T*_1_ numerically for a specific range of *T*_1_ values to calculate the maps. Several parameter combinations can yield equivalent *T*_1_ maps, provided that the appropriate expression for *ρ* (or a suitable approximation) is used, and specific requirements on such parameters are met, as detailed in [5].

The same equations can also be used to show that the (absolute) signal level of the image volume recorded at *T*_*I*,2_, being acquired later on the recovery curve, is generally larger than the one recorded at *T*_*I*,1_, in agreement with experimental observations. Hence, the second inversion image volume was employed for obtaining $T_{2}^{*}$ and *χ* estimates throughout this work. Instead, the *T*_1_ estimates were always obtained from the inversion contrast recorded with the first echo.

### 2.2 Simulation Studies

In order to evaluate the sensitivity of the ME-MP2RAGE signal to $B_{1}^{+}$ variations, we used simulations based on the Bloch equations. In particular, we plotted the signal expression *ρ* as a function of *T*_1_ with additional consideration of ±20% and ±40% variations in $B_{1}^{+}$ (through the flip angles). The convergence of the $B_{1}^{+}$ varied curves to the original signal expression indicates insensitivity to inhomogeneity of the $B_{1}^{+}$ field.

Because of the well-known $B_{1}^{+}$ inhomogeneity at 7 T, the nominal flip angle, *α*_*nom*_, may differ significantly from the effective flip angle, *α_eff_*, generated experimentally in a tissue. For more realistic comparisons, the flip angle values of the simulations and the experiments were matched using the efficiency factor *η_α_* ≡ *α_nom_* / *α_eff_*, where *α_eff_* was determined experimentally using $B_{1}^{+}$ maps.

The (ME-)MP2RAGE sequence yields *T*_1_ maps through an inversion-recovery acquisition where a mono-exponential recovery constant is estimated from two support points, *T*_*I*,(1,2)_, by inverting the signal expression for *T*_1_. Similarly, $T_{2}^{*}$ maps are obtained via a fitting procedure, where a mono-exponential decay constant is interpolated on ME measurements.

It is possible to investigate, through Monte-Carlo-like simulations, with the addition of noise [36], how the Signal-to-Noise Ratio (SNR) and the number and distribution of the support points affect the quality of the relaxation constant estimates. In particular, *n*_*T*_ = 100 different relaxation time values from the intervals *T*_1_/ ms ∈ [500, 3500] and $T_{2}^{*}/\;\text{ms}\in \left[2,60\right]$ were assumed, and up to *N* = 20000 simulations of the obtained signal levels were performed for each value with random variation of the noise contribution to compute standard deviations (SD), *σ*_*T*_, of the relaxation-time estimates. Based on typical experimental results, the SNR was set to 25, 50, or 100, with *T*_*I*,1_/ ms ∈ [500, 1500] and *T*_*I*,2_/ ms ∈ [1500, 3500] (constrained to *T*_*I*,2_ − *T*_*I*,1_ ≥ 1000 ms) in simulations of *T*_1_ mapping, and with *T*_*E*,*i*_/ ms ∈ [2, 30] and *n*_*E*_ ∈ [3, 5] in simulations of $T_{2}^{*}$ mapping (*n*_*E*_ is the number of echoes). The mean and SD of the *σ*_*T*_ values for the full range of relaxation times, *μ*_*σ*_ and *σ*_*σ*_, were then used to assess the robustness of the estimation.

Unless otherwise noted, simulations, data analyses (see below), and the visualization of the results were performed using the “Scientific Python” ecosystem [37–39].

### 2.3 Acquisition Parameter Considerations

This work targets at acquiring multiple quantitative maps of the full brain at sub-millimeter resolution with an acquisition time similar to current clinical practice, which implies that only a restricted set of acquisition parameter values are desirable or accessible. In order to achieve an advantage from the simultaneous mapping approach, the following conditions should be met: *(i)* the *T*_1_ map obtained with ME-MP2RAGE should be as accurate as a corresponding map acquired with MP2RAGE, and *(ii)* the acquisition time required for ME-MP2RAGE should be shorter than the sum of the acquisition times of corresponding MP2RAGE and ME-FLASH scans.

We specifically considered the following influences from the most relevant acquisition parameters: *(a)* lower values of *α*_1,2_ translate into improved $B_{1}^{+}$ insensitivity but also lower SNR, which directly affects the accuracy and precision; to maximize SNR, *α*_2_ was set to the Ernst angle, assuming *T*_1_ ≈ 1.6 s as the mean of the expected values for WM (≈ 1.2 s) and GM (≈ 2.0 s) [10]; *(b)* the number of *k*-space lines per GRE block affects the block duration and limits the range of possible inversion times; *n* increases with matrix size and Field Of View (FOV) and may be decreased through Parallel Acquisition Techniques (PAT); *(c)* the choice of *T*_*I*,(1,2)_ has an effect on the $B_{1}^{+}$ sensitivity and the accessible value range for *T*_1_ estimates as well as its precision; *(d)* the repetition time of the sequence, *T*_*R*,*seq*_, defines the total acquisition time and also has an (indirect) impact on the sensitivity to $B_{1}^{+}$ variation; *(e)* a short first *T*_*E*_ is desirable to maximize the SNR for *T*_1_ mapping; however, a relatively long (later) *T*_*E*_ in the order of 15 ms is useful for *χ* mapping (at the cost of longer *T*_*R*,*GRE*_ with concomitant constrains for matrix size and FOV) as it increases the Contrast-to-Noise Ratio (CNR) of the phase images and avoids possible anisotropy bias in the WM caused by non-linear phase evolution at short *T*_*E*_ [25, 26]; finally, acquiring more echoes improves $T_{2}^{*}$ mapping but might require a larger bandwidth, Δ*ν*.

The relatively large number of parameters (and, hence, optimization criteria) and their complex relations makes it impossible to define an *unbiased* cost function that simultaneously maximizes the accuracy and precision of all maps.

For this reason, a manual optimization approach was adopted: initially, the FOV, matrix, and PAT parameters were defined to determine *n*; then, we accommodated the maximum number of echoes within a fixed *T*_*R*,*GRE*_, long enough for reliable $T_{2}^{*}$ and *χ* mapping; lastly, we adjusted all other parameters to achieve sufficient $B_{1}^{+}$ insensitivity, and *T*_1_ mapping accuracy with the minimum possible *T*_*R*,*seq*_.

**Fig 2:**
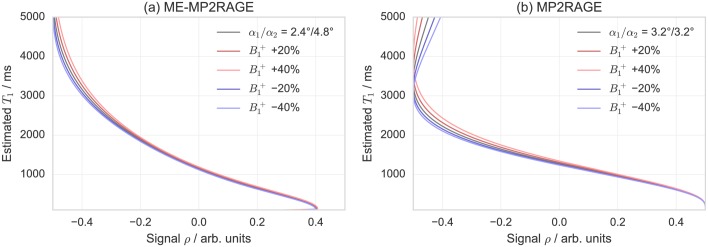
Estimated *T*_1_ values as a function of (a) the ME-MP2RAGE and (b) the MP2RAGE signal intensity parameter *ρ*. The green lines correspond to the effective acquisition parameters while red/blue lighter/darker lines indicate, respectively, ±20% and ±40% offsets of $B_{1}^{+}$. The flip angle values are adjusted for the accuracy factor *η*_*α*_. Note that the estimated *T*_1_ is limited to an upper value of approximately 3 s for the acquisition parameters that were used for MP2RAGE, which would result in *T*_1_ values exceeding this limit (e.g., in CSF) to be underestimated.

For the MP2RAGE acquisitions, a similar optimization approach was used, since the values suggested by [5] were not optimal for the desired resolution and FOV. In this case, the shorter *T*_*R*,*GRE*_ (due to *n*_*E*_ = 1) allowed to increase *n*, which was exploited to decrease the acquisition time. This was achieved both by shortening *T*_*R*,*seq*_ and by switching the first and second phase-encoding directions, so that the main (*T*_*R*,*seq*_) loop is performed along the GRAPPA-accelerated direction. However, in this case, the obtained acquisition parameters cause a systematically lower and potentially ambiguous *T*_1_ estimate in the CSF value range, which is not relevant in most applications. Note that this feature is intrinsic to the MP2RAGE-based *T*_1_ estimation procedure (see also: Fig 2) and the acquisition parameters should be adjusted to avoid this issue in the interval of interest.

Exploratory measurements (summarized in the Supporting Information) with a large coverage of different acquisition parameters were used to infer their effects, in relation to the corresponding simulations. The manually chosen parameters were then tested on a cohort of subjects to provide additional experimental evidence (allowing for group statistics).

### 2.4 MRI Experiments

Quantitative 3D MRI of the brain was performed in 19 healthy subjects (9 females, 23–31 years) on a Magnetom 7T scanner (Siemens Healthcare, Erlangen, Germany).

This study was approved by the Ethics Committee at the Medical Faculty of the University of Leipzig (application n.: 273-14-25082014). All participants had given informed written consent prior to the examination.

The ME-MP2RAGE pulse sequence was provided by Siemens. The acquisitions were performed using a monopolar readout (to avoid potential effects related to a constant offset in the gradients system), Generalized auto-calibRating Partially Parallel Acquisitions (GRAPPA) along the first phase-encoding (PE) direction, and 6/8 partial Fourier factors (both for the first and the second PE direction). The *in vivo* experiments were divided into two studies.

In *Study 1*, exploratory measurements were performed with a circularly-polarized transmit/24-channel receive head coil and a 32-channel receive version of the same coil design (Nova Medical, Wilmington, MA, USA) to evaluate the following range of acquisition parameters: FOV between 224×224×145.6 mm^3^ and 192×168×112 mm^3^ with nominal isotropic resolutions in the 0.6–0.9 mm range; GRAPPA acceleration factors, *f* = 2–3; *T*_*R*,*seq*_ = 4700–8000 ms; *T*_*I*,(1,2)_ = 750–1100, 2750–3500 ms; *α*_1,2_ = 2–5, 3–10 deg; *T*_*E*_ = 2.32–23.05 ms; *n*_*E*_ = 3–6; and Δ*ν* = 280–500 Hz/px yielding total acquisition times, *T*_*acq*_ = 5:55–22:25 min. MP2RAGE and ME-FLASH data were also recorded for the validation of the results with acquisition parameters similar to previously published values [4–6] or in-house standard settings. The FOV, nominal resolution, and GRAPPA acceleration were matched to the specific ME-MP2RAGE acquisition; further parameters were: for MP2RAGE *T*_*R*,*seq*_ = 5000–8000 ms; *α*_1,2_ = 4–5, 3–10 deg; *T*_*I*,(1,2)_ = 800, 2400 ms; *T*_*E*_ = 2.15 ms; Δ*ν* = 280 Hz/px; *T*_*acq*_ = 9:42 min; and for ME-FLASH *T*_*R*_ = 31–35 ms; *α* = 10–11 ms; *T*_*E*_ = 2.94–29.59 ms; *n*_*E*_ = 5–6; Δ*ν* = 280–500 Hz/px; *T*_*acq*_ = 6:04–12:40 min.

From the exploratory results, a preferred set of ME-MP2RAGE parameters (see Table 1) was derived to test, in *Study 2*, the intra-subject reproducibility with a FOV of 192 × 144 × 134.4 mm^3^ (axial orientation, first PE direction from right to left, second PE with 14% oversampling) and an acquisition matrix of 320 × 280 × 224 (i.e., 0.6 mm isotropic nominal resolution).

**Table 1:** Details of the manually optimized acquisition parameters that were used for the simulations in Fig 2 and for the acquisitions of *Study 2* with a nominal spatial resolution of 0.6 mm (isotropic).

Sequence	*T*_*R*,*seq*_	*T*_*I*_	*α*_*nom*_	*T*_*R*,*GRE*_	*n*	*n*_*E*_	*T*_*E*,1_	Δ*T*_*E*_	Δ*ν*	*T*_*acq*_
	[s]	[s]	[°]	[ms]	[#]	[#]	[ms]	[ms]	[Hz/px]	[min:s]
ME-MP2RAGE	6.0	0.75^a^	4	18.1	114	4	2.35	4.14	280	19:14
		2.90^b^	6							
MP2RAGE	5.0	0.80^a^	4	5.9	105	1	2.35	—	280	9:42
		2.40^b^	4							
ME-FLASH	—	—	11	31.0^c^	—	5	3.00	6.00	200	11:32

^a^ First GRE block.

^b^ Second GRE block.

^c^ *T*_*R*,*GRE*_ = *T*_*R*_ for (ME-)FLASH.

These scans were recorded with the 32-channel coil on 7 of the 19 subjects. To obtain reference data, further measurements were made in the same sessions with MP2RAGE and ME-FLASH (parameters included in Table 1) using the same FOV, matrix size, orientation, GRAPPA acceleration, and partial Fourier settings.

A typical session, thus, consisted of two MP2RAGE, two ME-FLASH and two ME-MP2RAGE acquisitions acquired in random order without repositioning.

In some sessions, additional $B_{1}^{+}$ maps were acquired with 3 mm isotropic nominal resolution employing an in-house modification (based on complex instead of magnitude images) of the Actual Flip-angle Imaging (AFI) technique [40]. Due to time restrictions, these maps were acquired in place of an ME-FLASH or MP2RAGE scan; thus, in such cases, ME-FLASH or MP2RAGE reproducibility results are not available.

### 2.5 Image Processing

*T*_1_ maps were reconstructed on-line by algorithms integrated in the Image Calculation Environment (ICE) provided by the vendor. $T_{2}^{*}$ maps were reconstructed off-line by log-linear least-squares fitting to the signal magnitudes, using the standard polynomial fit approach based on singular value decomposition. The QSM analysis was implemented in C++ using the ODIN library [41]. Susceptibility mapping was based on the phase of the echo acquired at the longest *T*_*E*_ for ME-MP2RAGE (*T*_*E*_ = 14.77 ms), and a similar value (*T*_*E*_ = 15.00 ms) was used for the ME-FLASH reference scans in order to improve comparability. The phase images were unwrapped using a Fourier method [42], which offers the advantage of describing singularities as continuous functions, and high-pass filtered using the Sophisticated Harmonic Artifact Reduction for Phase data (SHARP) approach [43]. Filtered phase images were converted into units of ppb (division by 10^−9^ *γ* *T*_*E*_ *B*_0_; *γ* is the gyromagnetic ratio of the proton), and quantitative maps were calculated with the Superfast Dipole Inversion (SDI) method [44].

Image segmentation and registration of brain tissues were accomplished using the FMRIB Software Library (FSL), Ver. 5.0 [45]. For each subject, a brain mask was generated (from the GRE readout at *T*_*I*,2_ for MP2RAGE or ME-MP2RAGE) using the Brain Extraction Tool (BET2) (with the ‘robust brain center estimation flag’ [-R] active), a Gaussian filter on BET’s result (for smoother edges) with SD *σ* = 0.5 px, and thresholding for values above 5% of the maximum. The extracted masks were furthermore shrunk (to avoid border effects) by the application of a binary erosion filter with a “spherical” kernel of diameter 1 px, iterated five times to remove the outer layer of CSF. Maps were then linearly co-registered using the FMRIB Linear Image Registration Tool (FLIRT) with rigid-body transformations, the correlation ratio metric and the previously obtained mask as weights. Once the volumes were registered, the non-brain tissues were masked out.

### 2.6 Statistical Analyses

Results obtained with ME-MP2RAGE were compared against MP2RAGE as reference for *T*_1_ maps and against ME-FLASH as reference for $T_{2}^{*}$ and *χ* maps. The equivalence of the maps was evaluated voxel-by-voxel with two-dimensional (2D) correlation histograms and difference images. For the quantification of correlations and mutual consistency of the maps, the following parameters were investigated:

1. The squared Pearson’s correlation coefficient, *r*^2^, as an indication of how well the test measurements reproduce the reference measurements (neglecting the noise of the reference measurements).
2. The means and SDs of the image volumes’ difference, as an estimate of respectively accuracy and precision,
  $$\mu_{D}=\frac{1}{N}\sum_{i=1}^{N}\left(y_{i}-x_{i}\right)\;\text{and}\;\sigma_{D}=\sqrt{\frac{1}{N}\sum_{i=1}^{N}{\left(\left(y_{i}-x_{i}\right)-\mu_{D}\right)}^{2}},$$ (4)
  as well as their absolute difference,
  $$\mu_{\left|D\right|}=\frac{1}{N}\sum_{i=1}^{N}|y_{i}-x_{i}|\;\text{and}\;\sigma_{\left|D\right|}=\sqrt{\frac{1}{N}\sum_{i=1}^{N}{\left(|y_{i}-x_{i}|-\mu_{D}\right)}^{2}},$$ (5)
  where *x*_*i*_ and *y*_*i*_ are defined by the map intensity of a voxel in the test data and in the reference data, respectively, and *N* is the number of voxels after the masking step. Note that *μ*_|*D*|_ and *σ*_|*D*|_ are equivalent (except for a factor $\sqrt{2}$) to the average and SD of the Euclidean distance from the identity line in the 2D histogram, and reflect the combined effects of deviation from the identity line and (random) spreading.

While *r*^2^ is often used in correlation studies, it only reflects potential deviations from a linear relationship, but it is not sensitive to the slope of the linear component of the relationship. It is thus of limited relevance if the measured values should be, ideally, identical as in the current case. As *r*^2^ is dimensionless, it more directly permits inter-modality comparisons. The accuracy and the precision of the measurement can be estimated by *μ*_*D*_ and *σ*_*D*_, respectively (in units of the quantity being measured). Additionally, if the accuracy is good, that is when *μ*_*D*_ ≈ 0, then *μ*_|*D*|_ (along with *σ*_|*D*|_ as its error) may be used as an estimate of the overall reproducibility. A sensible reference for these parameters is the size of the value range of the obtained maps. Note that these parameters are quite sensitive to outliers and may vary considerably depending on the efficacy of some of the procedures used in this work (e.g., the brain tissue masking, the fits used to calculate the maps, the registration, the considered value range, etc.).

To investigate the effects of potential errors arising from the registration procedure, we performed the voxel-by-voxel analysis described earlier on a given map in comparison to the same map after application of a linear rigid transformation. More specifically, translations in axial direction were examined for voxel offset values between 0.0 and 1.0 px; rotations about the axial direction and centered in the geometrical center of the FOV were examined for rotation angles between 0.0 and 1.0°; and rotations followed by translations were examined within the same value ranges. A complementary estimate of the effects of the registration step was obtained by applying the usual voxel-by-voxel analysis to “self-registered” maps. More precisely, reference maps were compared with the same maps registered back onto themselves after the application of a significant rigid transformation (in this study, a 10° rotation). These analyses were performed for *T*_1_ maps obtained with MP2RAGE and for $T_{2}^{*}$ and *χ* maps obtained with ME-FLASH.

Because of the relatively complex reconstruction technique required for QSM, it is not possible to use an equivalent simulation approach as for relaxometry to study the impact of the SNR. Nevertheless, it is possible to investigate the effects of SNR on the final QSM result by using an approach similar to what was done to investigate the effects of mis-registration. Particularly, we reconstructed several QSM maps obtained from the phase of the ME-FLASH complex images with the addition of Gaussian noise (with increasing SD in a range of 0–30 arb.units, corresponding to up to ≈ 100% decrease in SNR). The effect of the SNR on the *χ* maps was evaluated using the same voxel-by-voxel comparison analyses described earlier.

Additionally, we performed an analysis on a Region-Of-Interest (ROI) basis, where we calculated the group average parametric values across the different brain areas: frontal lobe (Fro.), temporal lobe (Tem.), parietal lone (Par.), occipital lobe (Occ.), insula (Ins.), putamen (Put.), caudate (Caud.), thalamus (Tha.), cerebellum (Cereb.) as defined by the MNI Structural Atlas. Additionally, white matter (WM) from the Harvard-Oxford Subcortical Structural Atlas was considered. Volumes from the second inversion images of the ME-MP2RAGE data were registered non-linearly to the MNI152 1 mm nominal resolution template (using default options) and such transformations were then used to bring the maps to the same space. Then, for each area (defined by the probabilistic masks thresholded for above 80%, which may incidentally leave out some portions of the WM), all the subjects from *Study 2* were group-averaged, and the group SD was also calculated.

## 3 Results

### 3.1 Simulations

**Fig 3:**
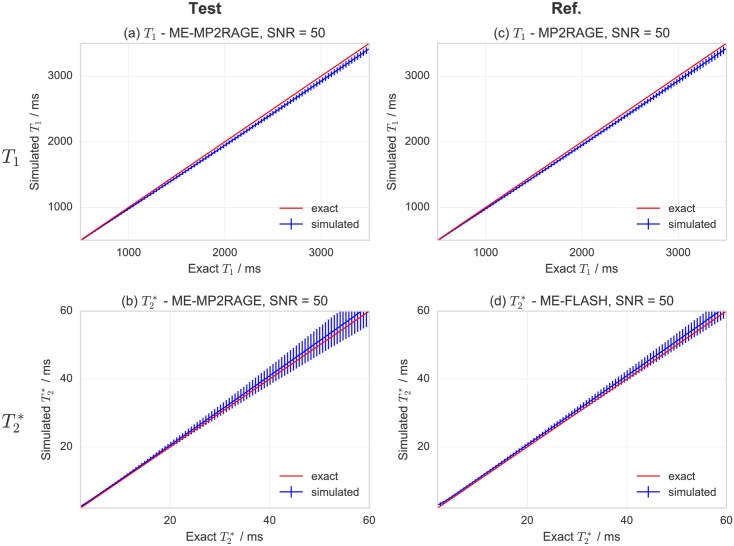
Simulations for the expected accuracy and precision of*T*_1_ and $T_{2}^{*}$ maps at SNR = 50. Rows refer to *T*_1_ (a, b) and $T_{2}^{*}$ (c, d) simulations, while columns indicate the choice of the simulation parameters reflecting the acquisition: ME-MP2RAGE (a, c), MP2RAGE (b) or ME-FLASH (d). Each panel contain a plot of the estimated relaxation time as a function of the exact value, with the error bars indicating the standard deviations, *σ*_*T*_, for *N* = 20000 simulations. The distance from the identity line indicates the expected accuracy, while the size of the error bars reflect the expected precision. For *T*_1_ / exponential recovery simulations, the range 0.5–3.5 s was probed, and the exact acquisition parameters simulated were *T*_*I*,(1,2)_ = 800, 2400 ms for ME-MP2RAGE, and *T*_*I*,(1,2)_ = 750, 2900 ms for MP2RAGE. For *T*_2_ / exponential decay simulations, the range 2–60 ms was probed, and the exact acquisition parameters simulated were *n*_*E*_ = 4, *T*_*E*,1_ = 2.5 ms, Δ*T*_*E*_ ≈ 4.2 ms for ME-MP2RAGE, and *n*_*E*_ = 5, *T*_*E*,1_ = 3.0 ms, Δ*T*_*E*_ = 6.0 ms for ME-FLASH.

The results obtained by relaxometry simulations (an overview of which is given in S3 and S4 Tables with additional details reported in S1 and S2 Figs) indicate that, for a given SNR, the number of echoes and their distribution is crucial for $T_{2}^{*}$ mapping, while the specific choice of inversion times (for the range examined in our study) is less critical for *T*_1_ mapping. As a rule of thumb, choices of inversion times according to *T*_*I*,1_/2 < *T*_1_ < 2*T*_*I*,2_ and echo times according to $T_{E,1}<T_{2}^{*}<T_{E,max}$ (where *max* indicates the longest *T*_*E*_) yield robust estimates of *T*_1_ and $T_{2}^{*}$, respectively, unless the SNR is insufficient. Detailed results for the specific settings of *T*_*I*_ and *T*_*E*_ that were selected for *Study 2* are shown in Fig 3. They indicate that the accuracy and precision (represented graphically by the distance from the identity line and by the size of the error bars, respectively) depend on the exact value of the relaxation times, and at this level of SNR are comparable between the test (ME-MP2RAGE) and the reference (MP2RAGE for *T*_1_, ME-FLASH for $T_{2}^{*}$) sequences. Additional information on the SNR dependence can be found in the support material indicated above.

**Fig 4:**
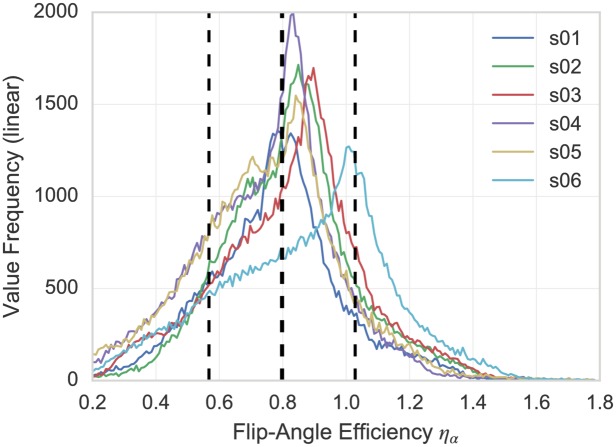
$B_{1}^{+}$ inhomogeneity displayed as histograms of the flip-angle accuracy factor inside the brain of six healthy human volunteers. Voxels outside the head or not containing brain tissue were masked out. The dashed lines indicate the mean (thick line) plus/minus the standard deviations (thin lines) of *η*_*α*_ across subjects.

Fig 4 shows combined histograms of the flip-angle accuracy factor measured in the brain with the 32-channel coil. The mean plus/minus SD were 0.8 ± 0.2. Hence, flip angle values used in the simulations were divided by 0.8 and rounded to the nearest integer to obtain corresponding settings for *α*_*nom*_ in the experimental protocols.

The variation of simulated intensity parameters *ρ* of the (ME-)MP2RAGE signal in dependence of *T*_1_ is presented in Fig 2. The underlying acquisition parameters are those from Table 1. These graphs were used to visually inspect the $B_{1}^{+}$ sensitivity and the acquisition parameters were selected accordingly. The level of the steady-state magnetization $M_{z}^{ss}$ is the major factor affecting $B_{1}^{+}$ sensitivity, while the accessible *T*_1_ range is mostly influenced by the specific choice of *α*_1,2_ and *T*_*I*,(1,2)_. Therefore, the number of *k*-space lines acquired in each GRE block, *n*, and *T*_*R*,*GRE*_, which relate directly to the image resolution and SNR, have a stronger impact on the $B_{1}^{+}$ sensitivity when the inter-acquisition times *T*_*A*_, *T*_*B*_, and *T*_*C*_ are shorter. Therefore, a relatively long *T*_*R*,*seq*_ is often needed for higher accuracy and robustness.

### 3.2 MRI Experiments

The results from the exploratory *Study 1* (summarized in the S5–S7 Tables) were used to investigate the impact of the acquisition scheme on the mapping. Further results obtained with a nominal resolution of 0.6 mm will be discussed in greater detail below. For higher resolutions, the statistical analyses did not indicate a strong impact from the particular imaging sequence. At lower resolutions, *T*_1_ maps obtained with ME-MP2RAGE and MP2RAGE were also essentially identical, whereas $T_{2}^{*}$ and *χ* maps obtained with ME-FLASH seemed slightly more accurate than those obtained with ME-MP2RAGE. This counter-intuitive observation is explained by the different PE schemes used in ME-MP2RAGE acquisitions with lower (e.g., 0.9 mm with first PE direction from head to foot) or higher (e.g., 0.6 mm with first PE direction from left to right) nominal resolutions, which yielded stronger restrictions in the choice of *T*_*E*,*max*_ (and, hence, less accurate $T_{2}^{*}$ fits) for the lower-resolution scans.

**Fig 5:**
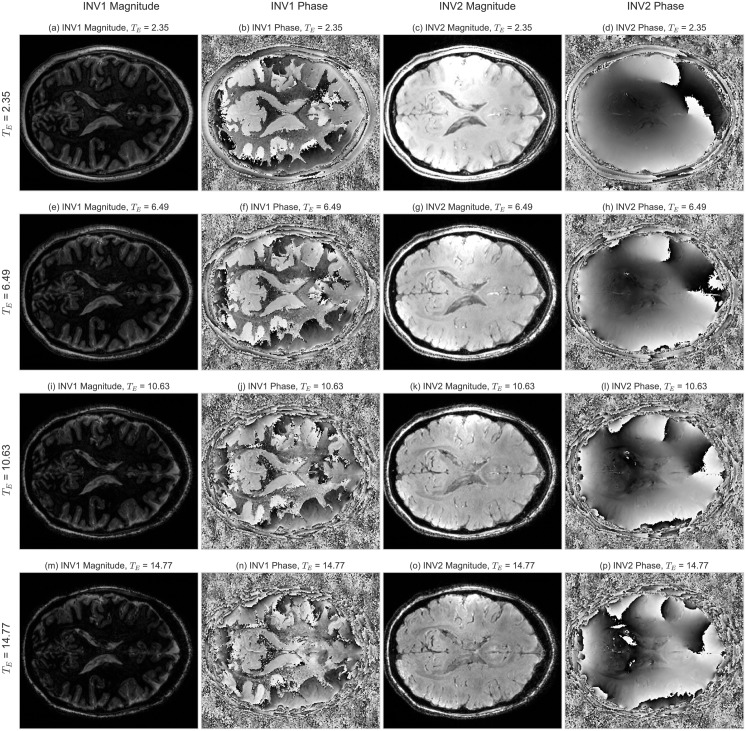
Example of the images obtained from an ME-MP2RAGE acquisition. Each row represents a different echo time. The columns show in order: first inversion magnitude (1st) and phase (2nd); second inversion magnitude (3rd) and phase (4th). Magnitude images are shown in arb.units, while phase image are in radians, both using a gray scale. Note that: *(i)* the phase images for the first inversion point show an abrupt change in their value corresponding to the zero crossing of the signal in the *T*_1_ recovery curve; *(ii)* the phase images for the second inversion point present some coil combination pole artifacts resulting in a corresponding degradation of the QSM maps at these locations.

Based on the combined results from the simulations and *Study 1*, the parameters in Table 1 were defined for an in-depth evaluation of data acquired with a high nominal resolution of 0.6 mm. An example of the data obtained with the ME-MP2RAGE sequence is shown briefly in Fig 5.

**Fig 6:**
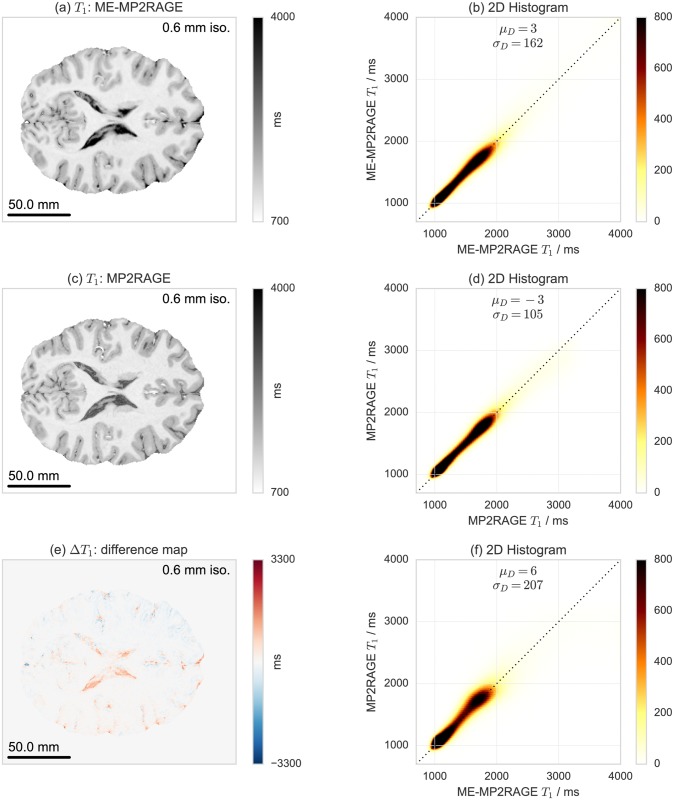
Single subject acquisition of *T*_1_ maps acquired with (a) ME-MP2RAGE, (c) MP2RAGE and (e) their difference, and test-retest reproducibility evaluation with voxel-by-voxel correlation 2D histograms for (b) ME-MP2RAGE, (d) MP2RAGE, and (f) ME-MP2RAGE versus MP2RAGE. Note how the CSF voxel are underestimated by the MP2RAGE as a result of the specific choice of the acquisition parameters.

**Fig 7:**
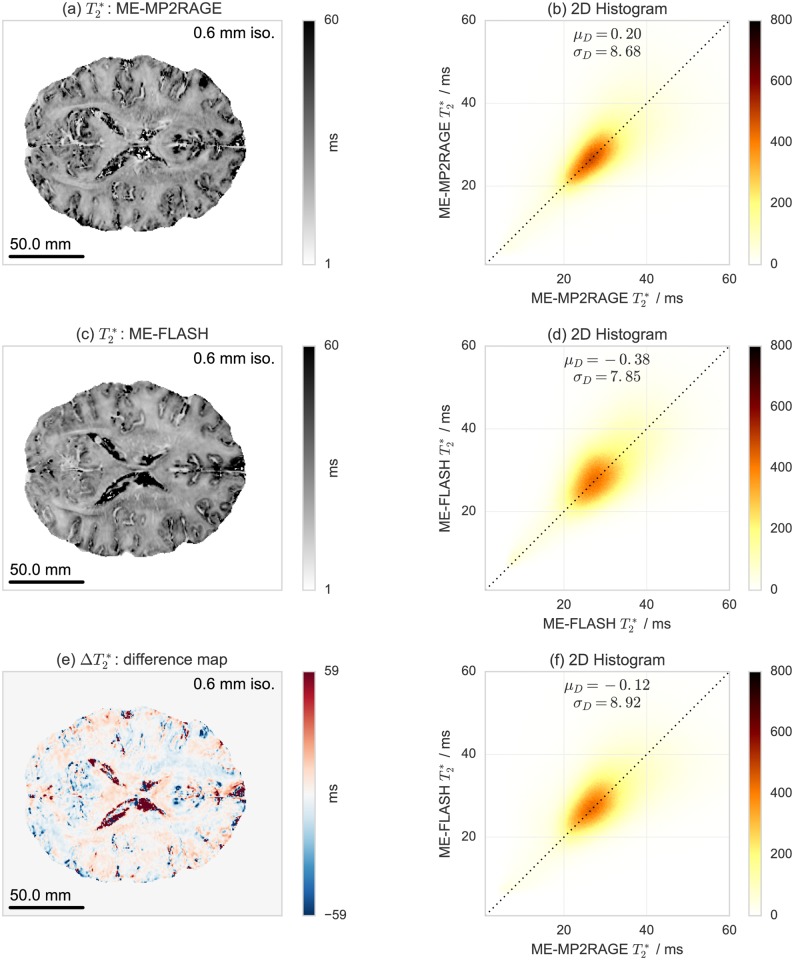
Single subject acquisition of $T_{2}^{*}$ maps acquired with (a) ME-MP2RAGE, (c) ME-FLASH and (e) their difference, and test-retest reproducibility evaluation with voxel-by-voxel correlation 2D histograms for (b) ME-MP2RAGE, (d) ME-FLASH, and (f) ME-MP2RAGE versus ME-FLASH.

**Fig 8:**
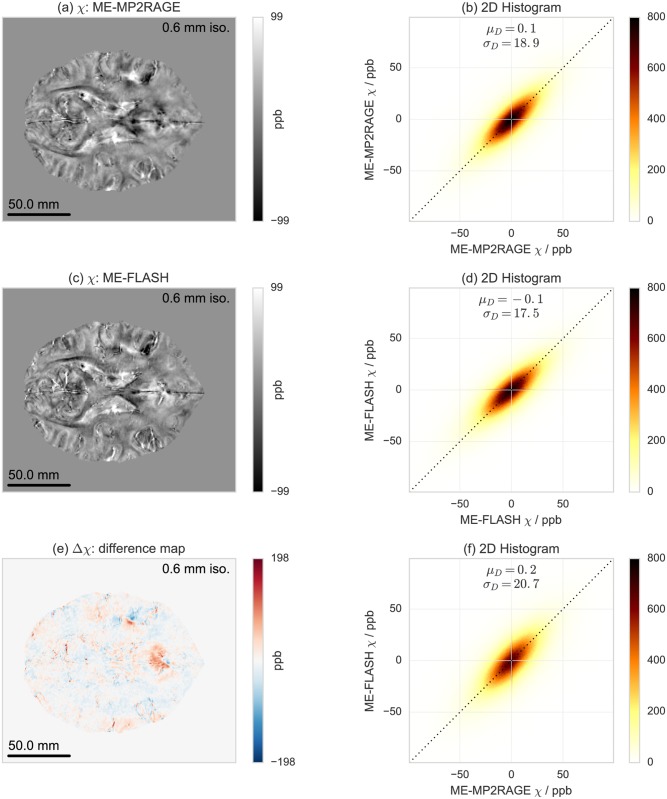
Single subject acquisition of *χ* maps acquired with (a) ME-MP2RAGE, (c) ME-FLASH and (e) their difference, and test-retest reproducibility evaluation with voxel-by-voxel correlation 2D histograms for (b) ME-MP2RAGE, (d) ME-FLASH, and (f) ME-MP2RAGE versus ME-FLASH.

A comparison of *T*_1_ maps acquired with ME-MP2RAGE and with MP2RAGE is shown in Fig 6. Similar comparisons of $T_{2}^{*}$ and *χ* maps derived with ME-MP2RAGE and with ME-FLASH are shown in Figs 7 and 8, respectively. These results are based on single subject acquisitions. Note that the *χ* maps are displayed in gray scale to be consistent with the QSM literature, but a diverging color map with linear luminance could be a better option for representing signed data (see also S8 Fig). The group statistics results are reported in Table 2. Individual subject results are reported in S8–S10 Tables.

**Table 2:** Summary of the group averages *μ*_*g*_ and SDs *σ*_*g*_ results of the correlation parameters for the *T*_1_, $T_{2}^{*}$ and *χ* maps acquired during *Study 2*.

Test	Ref.		*μ*_*D*_	*σ*_*D*_	*μ*_|*D*|_	*σ*_|*D*|_	*r*^2^
		*T*_1_	[ms]	[ms]	[ms]	[ms]	[#]
ME-MP2RAGE	MP2RAGE	*μ*_*g*_	11.9	141	94.6	105	0.873
		*σ*_*g*_	5.40	14.5	10.2	10.4	0.028
MP2RAGE	MP2RAGE	*μ*_*g*_	0.461	104	71.1	76.2	0.937
		*σ*_*g*_	7.59	12.9	8.20	9.98	0.018
ME-MP2RAGE	ME-MP2RAGE	*μ*_*g*_	1.01	108	72.4	80.5	0.917
		*σ*_*g*_	4.65	25.3	15.2	20.4	0.040
		$T_{2}^{*}$	[ms]	[ms]	[ms]	[ms]	[#]
ME-MP2RAGE	ME-FLASH	*μ*_*g*_	0.309	9.81	6.62	7.27	0.309
		*σ*_*g*_	0.625	1.29	0.985	0.872	0.087
ME-FLASH	ME-FLASH	*μ*_*g*_	0.0443	7.42	5.08	5.42	0.500
		*σ*_*g*_	0.273	1.15	0.923	0.729	0.114
ME-MP2RAGE	ME-MP2RAGE	*μ*_*g*_	0.459	10.3	6.85	7.68	0.329
		*σ*_*g*_	0.846	1.89	1.45	1.30	0.122
		*χ*	[ppb]	[ppb]	[ppb]	[ppb]	[#]
ME-MP2RAGE	ME-FLASH	*μ*_*g*_	−0.142	17.9	12.9	12.5	0.581
		*σ*_*g*_	0.139	3.44	2.59	2.28	0.140
ME-FLASH	ME-FLASH	*μ*_*g*_	0.141	16.3	11.8	11.3	0.654
		*σ*_*g*_	0.175	2.18	1.68	1.41	0.086
ME-MP2RAGE	ME-MP2RAGE	*μ*_*g*_	−0.0598	17.1	12.0	12.2	0.600
		*σ*_*g*_	0.0877	4.50	3.29	3.11	0.187

The 2D histograms of voxel-by-voxel correlations of *T*_1_ maps underline that the acquisition scheme produces very consistent results, both for within- as well as across-sequence comparisons. This is also supported by the mean and SDs of the image volumes’ differences, *μ*_*D*_ and *σ*_*D*_, being close to zero (relative to the *T*_1_ value range). However, some deviation is observed for very long *T*_1_ values corresponding to CSF voxels, as anticipated, because the selected MP2RAGE acquisition parameters were expected to systematically underestimate these values (see also Figs 2 and 6 and §2.3)

For the $T_{2}^{*}$ and *χ* maps, the 2D histograms and voxel-by-voxel correlations indicate that both ME-MP2RAGE and ME-FLASH do not achieve a similar degree of global reproducibility as observed for the *T*_1_ maps. While both $T_{2}^{*}$ and *χ* maps are essentially compatible across sequence types with *μ*_*D*_ values close to zero, the *σ*_*D*_ values are not negligible and, at least in the case of $T_{2}^{*}$, of almost the same order as the value range. Also, the bias introduced in the QSM results by the pole artifacts in the phase images seems to be independent of the acquisition scheme (see also S9 Fig). However, this does not seem to indicate inaccuracy of a specific acquisition scheme as the reproducibility does not substantially change for the within-sequence comparison. These results were consistently observed for all subjects.

The inter-acquisition averages of *μ*_*D*_, *μ*_|*D*|_, *σ*_*D*_, *σ*_|*D*|_ for the test ME-MP2RAGE acquisition, for the reference MP2RAGE and ME-FLASH acquisitions, and for the cross-comparisons are always within twice their respective SDs. Additionally, the *r*^2^ parameters for different acquisition schemes of the same mapping are always within a SD of each other. This suggests that the accuracy, precision and reproducibility of the mappings are only marginally dependent on the tested acquisition schemes.

The results from the ROI-based analysis for all maps are presented in Table 3.

**Table 3:** Group averages, *μ*_*g*_, and SDs, *σ*_*g*_, for the ROI-based analysis of ME-MP2RAGE acquisitions. Abbreviations: WM = White Matter; Fro. = Frontal Lobe; Tem. = Temporal Lobe; Par. = Parietal Lobe; Occ. = Occipital Lobe; Ins. = Insula; Put. = Putamen; Caud. = Caudate; Tha. = Thalamus; Cereb. = Cerebellum. Susceptibility is indicated here by Δ*χ* as a reminder of the values being referenced to an arbitrary offset, implying that the group average and SD values are expected to be biased by that and, possibly, by the pole artifacts of the phase images.

	WM	Fro.	Tem.	Par.	Occ.	Ins.	Put.	Caud.	Tha.	Cereb.
*T*_1_	[ms]	[ms]	[ms]	[ms]	[ms]	[ms]	[ms]	[ms]	[ms]	[ms]
*μ*_*g*_	1219	1802	1361	1541	1759	1592	1659	1441	1696	1546
*σ*_*g*_	30	51	39	65	73	54	36	34	44	75
$T_{2}^{*}$	[ms]	[ms]	[ms]	[ms]	[ms]	[ms]	[ms]	[ms]	[ms]	[ms]
*μ*_*g*_	25.85	33.1	23.7	30.3	37.9	29.76	33.2	27.4	30.7	31.5
*σ*_*g*_	0.80	1.7	1.8	1.4	2.2	0.83	1.9	1.1	1.9	1.5
Δ*χ*	[ppb]	[ppb]	[ppb]	[ppb]	[ppb]	[ppb]	[ppb]	[ppb]	[ppb]	[ppb]
*μ*_*g*_	7.0	-0.3	3.9	-0.8	1.7	-0.59	-0.4	26.3	-1.5	-0.36
*σ*_*g*_	2.3	1.0	5.5	1.3	5.4	0.12	1.4	3.4	1.6	0.22

### 3.3 Effects from Mis-registration and Noise

**Fig 9:**
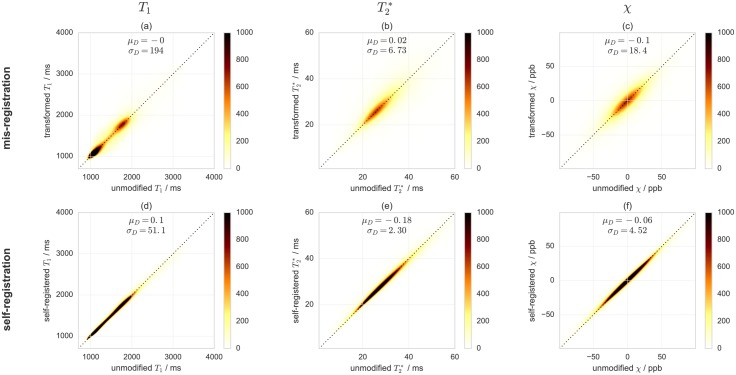
2D correlation histograms showing the effects of registration. Columns refer to *T*_1_ (a, c), $T_{2}^{*}$ (b, d) and *χ* (e, f) maps, while rows show either mis-registration (a, b, c) or self-registration (d, e, f) effects, respectively. The mis-registration and the self-registration effects are here illustrated by comparing each map with itself: after the application of a small transformation without registration (mis-registration) or after the application of a large transformation followed by a registration step (self-registration). The *x*-axis indicate the unmodified map, while the *y*-axis indicate the transformed or self-registered map. The small tranformation considered for the misregitration is a 0.5px translation followed by a 0.5° rotation. The large transformation considered for the self-registration is a 10° rotation.

The effects of registration are reported in Fig 9, where we show the 2D correlation histograms both on artificially mis-registratered and on self-registered *T*_1_, $T_{2}^{*}$, and *χ* maps, respectively. The original maps were obtained with MP2RAGE for *T*_1_ and with ME-FLASH for $T_{2}^{*}$ and *χ* in these cases. Additional results for correlation histograms and the statistical comparisons related to the mis-registration are provided in Simultaneous S3–S5 Figs and S11–S13 Tables, respectively.

The 2D histograms obtained from these analyses are comparable to those obtained from the comparison of different acquisitions, even for relatively small transformation parameters. These essentially capture the global effects of the considered transformations. Moreover, the correlation coefficients indicate different sensitivities for the different map types. Specifically, a relatively linear behavior of *r*^2^ for the explored range of parameters is found for *T*_1_ and *χ* maps, while there is a remarkably non-linear drop in *r*^2^ for the same range of transformations in the case of $T_{2}^{*}$ (e.g., *r*^2^ dropped from 0.952 to 0.918 for *T*_1_ and from 0.913 to 0.850 for *χ* but from 0.615 to 0.401 for $T_{2}^{*}$ in analyses of subtle translations of 0.375 px and 0.500 px). The comparisons of the “self-registered” maps indicate that the effect of the registration step on the correlation analyses is *at least* of the same order of magnitude of a 0.2° rotation, which is alone sufficient to explain an *r*^2^ of ≈ 0.99, 0.91 and 0.97 for *T*_1_, $T_{2}^{*}$ and *χ* respectively, the major effect again being observed with $T_{2}^{*}$ maps. Note that self-registration results indicate only a lower limit on the effects of the registration step because of the negative effects of noise. Additionally, neither *r*^2^ nor the other correlation parameters are linear with respect to SNR, mis-registration or their combination; therefore these results cannot be used to quantify the single effects.

The explored noise level range correspond to up to about half of the SNR of the original images. The values of the obtained coefficients indicate that such SNR reductions degrade the voxel-by-voxel correlations with an effect size that is comparable to that from a translation of approximately 0.5 px, a rotation by approximately 0.9°, or a 0.4° rotation followed by a 0.4 px translation. An example of the effects of an increased noise level on *χ* maps is reported in S6 Fig. A summary of the correlation analysis is provided in S14 Table.

## 4 Discussion

### 4.1 Acquisition Parameters

In this work, simulations and exploratory MRI experiments were employed to optimize an ME-MP2RAGE sequence modification for high-resolution simultaneous mapping of *T*_1_, $T_{2}^{*}$, and *χ*. The proposed set of acquisition parameters is specifically tailored to the tested resolutions and FOV. The software tools developed for this work can effectively be used for optimizing the acquisition parameters for different use cases. However, their limitations should be properly considered.

Particularly, the solutions of the Bloch equations for evaluating the $B_{1}^{+}$ sensitivity are ignoring the experimentally available SNR. This is of particular relevance when considering that the *T*_1_ maps are more insensitive to $B_{1}^{+}$ inhomogeneities at lower (relative) $M_{z}^{ss}$, which is typically achieved by lowering the flip angles *α*_1_ and *α*_2_ or increasing the *T*_*R*,*seq*_. However, this might have a negative impact on the acquisition time or the SNR (depending on *T*_*R*,*GRE*_, which in turn determines the Ernst angle).

The fitting procedures that were used to infer the influence of SNR and sampling points on the accuracy and precision of the estimated relaxation time constants (i.e., *T*_1_ from *T*_*I*,(1,2)_ and $T_{2}^{*}$ from different *T*_*E*,*i*_) are based on the implicit assumption that the signal is well modeled by a mono-exponential function. However, recent works suggest that more complex curves (e.g., multi-exponential) might be required for modeling both longitudinal [12] and transverse [25, 46, 47] magnetization behaviors. More elaborate acquisition schemes with many more support points would be required to further investigate this aspect, which is neglected at this level.

Additionally, while the physical experiment is fundamentally identical, the methods for obtaining *T*_1_ are slightly different for the simulation and the measurements. While the simulations rely on the fitting of magnitudes images, the measurements use a combination of the complex images to obtain the MP2RAGE signal which is then converted to *T*_1_ via a look-up table (see also: Fig 2). In fact, the MP2RAGE approach is more accurate [5], essentially because the phase images provide additional information, particularly for the first inversion image, where the magnitude-only images have very low intensity due to the proximity to the zero-crossing in the inversion recovery curve. Therefore, the simulation constitutes a lower limit (except for SNR considerations) to the accuracy of the *T*_1_ estimates.

The proposed procedure for the choice of the acquisition parameters requires a manual optimization, which inherently implies a certain degree of arbitrariness. While a more formal approach would be possible, there are several aspects that should be considered. Firstly, the arbitrariness is inherent to the problem, because a particular desired feature (e.g., resolution, accuracy, speed) is obtained at the expenses of the others. For example, it would be possible to write a cost function for $B_{1}^{+}$ insensitivity and a cost function for the coverage of a specific range of *T*_1_ values. However, there is no unbiased way of combining the two without introducing some degree of arbitrariness, and further weights would need to be integrated into the model in order to cover the most relevant aspects of the acquisition. Such weights might not have immediate physical links with the desired features, making it difficult to find a rationale for their definition. Secondly, given the structure of the manifold parameters to be explored, there exist several suboptimal combinations as already suggested by [5], and an optimization algorithm might not find the best solution. Although a metric could be defined to describe the marginal gain associated with slight changes in parameters (e.g., by taking the partial derivatives of “first order” cost functions with respect to the parameters optimized by the other cost functions), this would lead to a much more complex mathematical model and would introduce additional arbitrary weights. Lastly, given the limits to specify sequence parameters on the protocol level (e.g., the flip angle can only be set to integer values in our current implementation), an automated optimization procedure is likely to produce a set of parameters that is not accessible with the same exactness in an experimental situation. Ultimately, this does not justify the additional complexity of such an approach. For these reasons, we performed the simulations by choosing the parameters with a rationale based on provisions of the desired resolution and scan time. These results were then extensively tested experimentally, thus providing additional information on the impact of aspects that were neglected or only partially addressed by the simulations.

### 4.2 Simultaneous Parameter Mapping

Focusing on the voxel-by-voxel comparisons of results obtained with different or the same acquisition modalities, a major point to discuss is the validity of the method for assessing the impact of the acquisition technique on the map accuracy. Since there was no direct access to the “ground truth”, we assumed that a more-established acquisition technique can be used as a “gold standard” for a specific map. Based on this, we consider two different acquisition schemes to be experimentally equivalent if they have the same degree of reproducibility (e.g., similar *μ*_*D*_ and *σ*_*D*_) as two separate repetitions with the same sequence. This rationale relies on the assumption that the assessment of the reproducibility is unbiased. However, the registration step has an important effect on the parameters used to quantify reproducibility. This bias is always present (for non-simultaneous acquisitions), arising from the numerical interpolation required to address geometrical transformations acting on a sub-voxel scale—even in ideal cases where other spatial inaccuracies due to movements of the subject during the acquisition or noise associated with physiological processes could be ignored. While errors arising during registration are not sufficient to entirely explain the observed level of reproducibility, our simulation results are compatible with the hypothesis that even subtle mis-registrations are able to largely explain the values of the statistical coefficients used to assess the reproducibility. This is especially true for the measured $T_{2}^{*}$ (and, partially, also for *χ*) maps, because the inter-voxel values fluctuations are observed at a much finer spatial scale compared to the measured *T*_1_ maps. These differences are mostly localized at the boundaries of regions characterized by different average values (e.g. GM/WM, GM/CSF, etc.) and are sufficiently strong to be captured by the global coefficients and plots considered.

The lower reproducibility that is observed for the $T_{2}^{*}$ and *χ* (compared to *T*_1_) may not entirely be explained by registration issues, and further investigation may be required to elucidate other potentially relevant sources of error, for example physiological noise or motion. This would improve the understanding of the link between the tissue microstructure and $T_{2}^{*}$ mapping at a finer level. To reduce the effect of noise in these maps, it would be possible to introduce in the fit algorithm a regularization term incorporating additional information, for example from neighboring voxels [12, 46] or from less noisy acquisitions like the simultaneously measured *T*_1_.

Regardless of the acquisition scheme, our results also indicate a higher reproducibility of *χ* results compared to $T_{2}^{*}$. However, this result would need a more robust validation. In fact, the correlation coefficients considered in this work are not appropriate for accurate quantification of this aspect. Additionally, the QSM results are obtained on a smaller volume (due to limitations of the processing pipeline) and, hence, a comparison of both contrasts might be biased by this difference. Note also that $T_{2}^{*}$ values were generally longer in the voxels excluded for QSM and might be estimated with an increased error because the acquisition parameters had been optimized for values up to ≈ 45 ms.

The SNR available for $T_{2}^{*}$ and *χ* estimates was inherently lower for ME-MP2RAGE as compared to ME-FLASH with the tested parameters. This is because substantially longer *T*_*R*_ values and higher flip-angles were used for ME-FLASH. Additionally, the SNR of the second GRE block in (ME-)MP2RAGE is inversely modulated by *T*_1_ relaxation. Despite these differences in the SNR, the reproducibility coefficients are not appreciably different across acquisition schemes, suggesting that either the mapping or the comparison procedure (or both) are not sensitive enough for subtle SNR variations.

Of note, when choosing *T*_*E*_ for both ME-FLASH and ME-MP2RAGE, we ignored potential effects related to the phase difference between water and fat. This was not a significant issue because usually the fat contribution to the signal in brain tissues is negligible, since most lipid molecules are immobilized in membranes and will have a $T_{2}^{*}$ in the *μ*s range, which is too short to be observed with the typical echo times of a standard ME-FLASH experiment. Otherwise, we would have expected an acquisition-related bias in the $T_{2}^{*}$ maps, which would have caused, for example, the *μ*_*D*_ parameter between different acquisition to appreciably deviate from zero, and this was not observed.

The *T*_1_ values obtained from the ROI-based analysis indicate excellent agreement with [10] for WM, while GM values are consistently lower in our experiments. This might be explained by a slightly different definition of the ROIs and varying partial voluming effects, especially with CSF. $T_{2}^{*}$ values are generally in agreement with previously published results (e.g., [48, 49]), although it should be noted that $T_{2}^{*}$ results are always modulated—and therefore biased—by the *B*_0_ shimming. The susceptibility results are relative to an arbitrary reference, which was chosen to obtain an average susceptibility value very close to zero. For this reason, the group-averages in this case may be of limited significance.

In its current form, the ME-MP2RAGE pulse sequence achieves simultaneous acquisition of *T*_1_, $T_{2}^{*}$, and *χ* maps as a trade-off between resolution, SNR, and scan time. Our set of parameters (Table 1) showed that a slightly reduced SNR to stay within the constrained acquisition time was still sufficient for relatively accurate simultaneous mapping. A more significant impact on the map accuracy, however, is expected for more aggressive reductions of the scan time (e.g., by further increasing the GRAPPA factor). Instead, if the resolution and FOV constraints are relaxed, it is possible to reach the point where the MP2RAGE and its ME variant have identical scan times, but the latter allows the simultaneous acquisition of *T*_1_, $T_{2}^{*}$ and *χ* maps without a further penalty for the achieved accuracy. This situation might be more interesting for 3 T, where typical resolutions are lower. This aspect remains to be experimentally investigated, but the already mentioned simulation tools can be effectively used to predict the reliability of *T*_1_ estimates.

Further sequence developments may allow to address some of the current limitations of ME-MP2RAGE. A finer control over timing and *k*-space coverage will improve the flexibility of the sequence, thus allowing for better trade-offs in resolution, scan time, and maps accuracy. This could be achieved for example by mixing the first and the second phase encoding directions in each GRE block (eventually integrating compressed sensing techniques) and/or by using a different number (and eventually timings) for the acquired echoes across the GRE blocks, but this would require both major modification to the sequence code and careful consideration on the effects on the *T*_1_ point-spread function, and ultimately a separate study to evaluate its accuracy in estimating *T*_1_ maps.

Such modifications may also lead to the opportunity of acquiring more sampling points in the inversion recovery curve. This is interesting because it might offer an effective and time saving sequence for the purpose of investigating non-mono-exponential behaviors, and hence partial volumes effects.

Another approach toward the acquisition of multiple images at different inversion times was recently proposed with the MPnRAGE sequence [50], where a radial readout scheme is employed instead of the cartesian one.

## 5 Conclusion

The ME-MP2RAGE scheme with an appropriate choice of acquisition parameters permits the quantification of *T*_1_, $T_{2}^{*}$, and magnetic susceptibility *χ* *in vivo* at 7 T. The resulting maps are reasonably well comparable to results obtained from more-established techniques, such as standard MP2RAGE and ME-FLASH. The time required for recording multiple 3D maps simultaneously, even at high nominal resolution of 0.6 mm, is shorter than the time required to obtain corresponding maps from separate acquisitions. This aspect is even more favorable at lower resolution, where the timing is more flexible and the acquisition becomes more efficient with the current pulse-sequence design.

This sequence modification may allow researchers and clinicians to gain additional useful tissue information from a single experiment with improved consistency regarding subtle head motion and without a need for registration of image volumes with different contrast.

## Supporting Information

S1 Table List of Abbreviations. A list of common abbreviations used through the text.
(PDF)

S2 Table List of Symbols. A list of common symbols used through the text.
(PDF)

S1 Fig Dependece of *T*_1_ simulations from SNR. Plot of *T*_1_ estimates obtained with simulations versus the exact input values (range 0.5–3.5 s) as a function of the SNR. Error bars indicate the standard deviations, *σ*_*T*_, for *N* = 20000 simulations. Different columns correspond to different SNR levels of (a,d) 25, (b,e) 50, and (c,f) 100, while the two rows show the results for (a–c) ME-MP2RAGE with *T*_*I*,(1,2)_ = 800, 2400 ms and for (d–f) MP2RAGE with *T*_*I*,(1,2)_ = 750, 2900 ms.
(TIFF)

S2 FigDependece of $T_{2}^{*}$ simulations from SNR.Plot of $T_{2}^{*}$ estimates obtained with simulations versus the exact input values (range 2–60 ms) as a function of the SNR. Error bars indicate the standard deviations, *σ*_*T*_, for *N* = 20000 simulations. Different columns correspond to different SNR levels of (a,d) 25, (b,e) 50, and (c,f) 100, while the two rows show the results for (a–c) ME-MP2RAGE with *n*_*E*_ = 4, *T*_*E*,1_ = 2.5 ms, and Δ*T*_*E*_ ≈ 4.2 ms and for (d–f) ME-FLASH with *n*_*E*_ = 5, *T*_*E*,1_ = 3.0 ms, and Δ*T*_*E*_ = 6.0 ms.(TIFF)

S3 Fig 2D histograms showing mis-registration effects in *T*_1_ maps. The *x*-axis refers to the unmodified map, while the *y*-axis refers to the same map after application of: (a) a translation by 0.2 px, (b) a rotation by 0.2°, (c) a rotation by 0.2° followed by a translation by 0.2 px, (d) a translation by 0.5 px, (e) a rotation by 0.5°, (f) a rotation by 0.5° followed by a translation by 0.5 px. More details can be found in S11 Table.
(TIFF)

S4 Fig2D histograms showing mis-registration effects in $T_{2}^{*}$ maps.The *x*-axis refers to the unmodified map, while the *y*-axis refers to the same map after application of: (a) a translation by 0.2 px, (b) a rotation by 0.2°, (c) a rotation by 0.2° followed by a translation by 0.2 px, (d) a translation by 0.5 px, (e) a rotation by 0.5°, (f) a rotation by 0.5° followed by a translation by 0.5 px. More details can be found in S12 Table.(TIFF)

S5 Fig 2D histograms showing mis-registration effects in *χ* maps. The *x*-axis refers to the unmodified map, while the *y*-axis refers to the same map after application of: (a) a translation by 0.2 px, (b) a rotation by 0.2°, (c) a rotation by 0.2° followed by a translation by 0.2 px, (d) a translation by 0.5 px, (e) a rotation by 0.5°, (f) a rotation by 0.5° followed by a translation by 0.5 px. More details can be found in S13 Table.
(TIFF)

S6 Fig 2D histograms for *χ* maps showing effects from differing noise levels. The *x*-axis refers to the unmodified map, while the *y*-axis refers to a map calculated from the same source complex image after adding Gaussian noise with a SD of (a) 5, (b) 10, (c) 15, (d) 20, (e) 25, (f) 30 arbitrary intensity units, corresponding to up to 100% increase in SNR. The original SNR was estimated as the maximum of the magnitude image divided by the standard deviation of the real image in a region where no signal was expected. More details can be found in S14 Table.
(TIFF)

S7 Fig2D histograms for the self-registration effects.Results for (a) *T*_1_, (b) $T_{2}^{*}$ and (c) *χ* maps are shown. The *x*-axis refers to the unmodified map, while the *y*-axis refers to the self-registered map, i.e. the same map after the application of a 10° rotation and then registered back (using FLIRT) onto the original map.(TIFF)

S8 Fig Example of different color maps used for magnetic susceptibility mapping. The same magnetic susceptibility map is displayed with the standard gray scale color map (a), a diverging color map with non-linear (b) and (almost) linear luminance (c). The divergin color map with linear luminance (see also: http://matplotlib.org/users/colormaps.html and “A Better Default Colormap for Matplotlib.” Berkeley Institute for Data Science, July 17, 2015. https://bids.berkeley.edu/resources/videos/better-default-colormap-matplotlib) may be a better option (although not used in previous literature) because it allows for a clear separation of positive and negative values, while retaining as much quantitative information as a gray color scale. This is actually interesting in view of the applications where a separation from paramagnetic and diamagnetic substances is desired. On the other hand, the arbitrary reference currently limits its efficacy and its adoption could be postponed until the community reaches a consensus on this.
(TIFF)

S9 Fig Example of the consistency of pole artifact across acquisitions. Phase images from the same subject and session are shown side by side for the ME-MP2RAGE acquisition and the ME-FLASH acquisition. The position of the pole artifact is consistent between the two acquisition (within the limits of subject’s motion), thus suggesting a limited impact on test-retest reproducibility.
(TIFF)

S3 Table Simulation of SNR influence on *T*_1_ mapping. Average and standard deviation of *σ*_*T*_ values in simulations of *T*_1_ mapping (assumed *T*_1_ values between 0.5 and 3.5 s) to evaluate the SNR influence.
(PDF)

S4 TableSimulation of SNR influence on $T_{2}^{*}$ mapping.Average and standard deviation of *σ*_*T*_ values in simulations of $T_{2}^{*}$ mapping (assumed $T_{2}^{*}$ values between 2 and 60 ms) to evaluate the SNR influence.(PDF)

S5 Table Correlation parameters obtained in *Study 1* for *T*_1_ mapping. The order of the listed acquisition parameters is: nominal isotropic resolution, *T*_*R*,*seq*_, *α*_1,2_, *T*_*I*,(1,2)_, and *T*_*E*_.
(PDF)

S6 TableCorrelation parameters obtained in *Study 1* for $T_{2}^{*}$ mapping.The order of the listed acquisition parameters is: nominal isotropic resolution, *T*_*R*,*seq*_, *α*_1,2_, *T*_*I*,(1,2)_, and *T*_*E*_ values for ME-MP2RAGE; nominal isotropic resolution, *T*_*R*_, *α* and *T*_*E*_ values for ME-FLASH.(PDF)

S7 Table Correlation parameters obtained in *Study 1* for *χ* mapping. The order of the listed acquisition parameters is: nominal isotropic resolution, *T*_*R*,*seq*_, *α*_1,2_, *T*_*I*,(1,2)_, and *T*_*E*_ for ME-MP2RAGE; nominal isotropic resolution, *T*_*R*_, *α* and *T*_*E*_ for ME-FLASH.
(PDF)

S8 Table Statistical coefficients for *T*_1_ mapping from the different acquisitions. Variations of the correlation coefficients, and means and SDs of image volume differences (as defined in Eqs 4 and 5) obtained in *Study 2* for *T*_1_ maps across different subjects with the acquisition parameters from Table 1. The last part of the table are the group averages *μ*_*g*_ and SDs *σ*_*g*_ according to the acquisition scheme.
(PDF)

S9 TableStatistical coefficients for $T_{2}^{*}$ mapping from the different acquisitions.Variations of the correlation coefficients, and means and SDs of image volume differences (as defined in Eqs 4 and 5) obtained in *Study 2* for $T_{2}^{*}$ maps across different subjects with the acquisition parameters from Table 1. The last part of the table are the group averages *μ*_*g*_ and SDs *σ*_*g*_ according to the acquisition scheme.(PDF)

S10 Table Statistical coefficients for *χ* mapping from the different acquisitions. Variations of the correlation coefficients, and means and SDs of image volume differences (as defined in Eqs 4 and 5) obtained in *Study 2* for *χ* maps across different subjects with the acquisition parameters from Table 1. The last part of the table are the group averages *μ*_*g*_ and SDs *σ*_*g*_ according to the acquisition scheme.
(PDF)

S11 Table Statistical coefficients for *T*_1_ mapping from mis-registration. Variations of the correlation coefficients, and means and SDs of image volume differences (as defined in Eqs 4 and 5) obtained for systematic geometrical transformations of *T*_1_ maps.
(PDF)

S12 TableStatistical coefficients for $T_{2}^{*}$ mapping from mis-registration.Variations of the correlation coefficients, and means and SDs of image volume differences (as defined in Eqs 4 and 5) obtained for systematic geometrical transformations of $T_{2}^{*}$ maps.(PDF)

S13 Table Statistical coefficients for *χ* mapping from mis-registration. Variations of the correlation coefficients, and means and SDs of image volume differences (as defined in Eqs 4 and 5) obtained for systematic geometrical transformations of *χ* maps.
(PDF)

S14 Table Statistical coefficients for *χ* mapping from differing noise levels. Variations of the correlation coefficients, and means and SDs of image volume differences (as defined in Eqs 4 and 5) obtained for systematic increase of the noise level for the complex images used to compute *χ* maps.
(PDF)
