# Supplementary material for: Simultaneous Quantitative MRI Mapping of T1, T2* and Magnetic Susceptibility with Multi-Echo MP2RAGE
Source: PLoS One. 2017 Jan 12;12(1):e0169265. doi: 10.1371/journal.pone.0169265 (PMC5230783; doi:10.1371/journal.pone.0169265)
Supplement: S1 Table — A list of common abbreviations used through the text. (PDF) [file pone.0169265.s001.pdf]

2D, 3D	= two-, three-dimensional;
AFI	= actual flip-angle imaging;
BW	= bandwidth;
CNR	= contrast-to-noise ratio;
CSF	= cerebro-spinal fluid;
FLASH	= fast low-angle shot;
FOV	= field of view;
GM	= gray matter;
GRAPPA	= Generalized auto-calibRating Partially Parallel Acquisitions;
GRE	= gradient-recalled echo;
ME	= multi-echo;
MP2RAGE	= magnetization-prepared 2 rapid gradient echoes;
MP-RAGE	= magnetization-prepared rapid gradient echo;
MR	= magnetic resonance;
MRI	= magnetic resonance imaging;
QSM	= quantitative susceptibility mapping;
PAT	= parallel acquisition technique;
PE	= phase encoding;
RF	= radio-frequency;
SD	= standard deviation;
SNR	= signal-to-noise ratio;
WM	= white matter.
