# Supplementary material for: Simultaneous Quantitative MRI Mapping of T1, T2* and Magnetic Susceptibility with Multi-Echo MP2RAGE
Source: PLoS One. 2017 Jan 12;12(1):e0169265. doi: 10.1371/journal.pone.0169265 (PMC5230783; doi:10.1371/journal.pone.0169265)
Supplement: S2 Table — A list of common symbols used through the text. (PDF) [file pone.0169265.s002.pdf]

B_0	:	amplitude of the main magnetic field;
B_1^+	:	amplitude of the RF transmit magnetic field;
E_1	:	longitudinal relaxation factor for the period TR_{GRE};
E_A	:	longitudinal relaxation factor for the period T_A;
E_C	:	longitudinal relaxation factor for the period T_C;
f	:	GRAPPA acceleration factor;
i	:	index;
M_0	:	equilibrium magnetization;
$M_z(0^{\pm})$	:	longitudinal magnetization immediately after/before an RF pulse;
M_z^{ss}	:	steady-state longitudinal magnetization;
N	:	number of simulations or number of voxels after masking;
n	:	number of k-space lines acquired during one GRE block;
n_E	:	number of echoes;
n_T	:	number of relaxation time values;
r	:	Pearson's product-moment correlation coefficient;
S	:	signal voltage;
T_1	:	longitudinal relaxation time;
T_2^*	:	effective transverse relaxation time;
T_A	:	duration from the inversion pulse to the onset of the first GRE block;
T_{acq}	:	acquisition time;
T_B	:	duration from the end of the first to the onset of the second GRE block;
T_C	:	duration from the end of the second GRE block to the next inversion pulse;
T_E	:	echo time;
$T_{E,max}$	:	maximum echo time;
ΔT_E	:	inter-echo time;
T_I	:	inversion time;
$T_{R,GRE}$	:	repetition time within the GRE block;
$T_{R,seq}$	:	sequence repetition time as defined by the separation of inversion pulses;
x	:	variable;
x_i, y_i	:	intensities of the i-th voxel, resp. in the test map and the reference map;
α	:	RF pulse flip angle;
α_{eff}	:	effective flip angle;
α_{nom}	:	nominal flip angle;
γ	:	gyromagnetic ratio;
η	:	inversion efficiency;
η_α	:	flip-angle accuracy factor;
μ_D	:	mean of the image volumes' difference;
$\mu_{\|D\|}$	:	mean of the image volumes' absolute difference;
μ_g	:	group average;
μ_σ	:	mean of σ_T values;
$\Delta\nu$	:	receiver bandwidth;
ξ	:	scaling constant;
ρ	:	intensity parameter of the combined MP2RAGE images;
σ	:	standard deviation;
σ_D	:	standard deviation of the image volumes' difference;
$\sigma_{\|D\|}$	:	standard deviation of the image volumes' absolute difference;
σ_g	:	group standard deviation;
σ_T	:	standard deviation of a relaxation-time estimate;
σ_σ	:	standard deviation of σ_T values;
χ	:	bulk magnetic susceptibility.
