# Supplementary figures and images for: Simultaneous Quantitative MRI Mapping of T1, T2* and Magnetic Susceptibility with Multi-Echo MP2RAGE
Source: PLoS One. 2017 Jan 12;12(1):e0169265. doi: 10.1371/journal.pone.0169265 (PMC5230783; doi:10.1371/journal.pone.0169265)

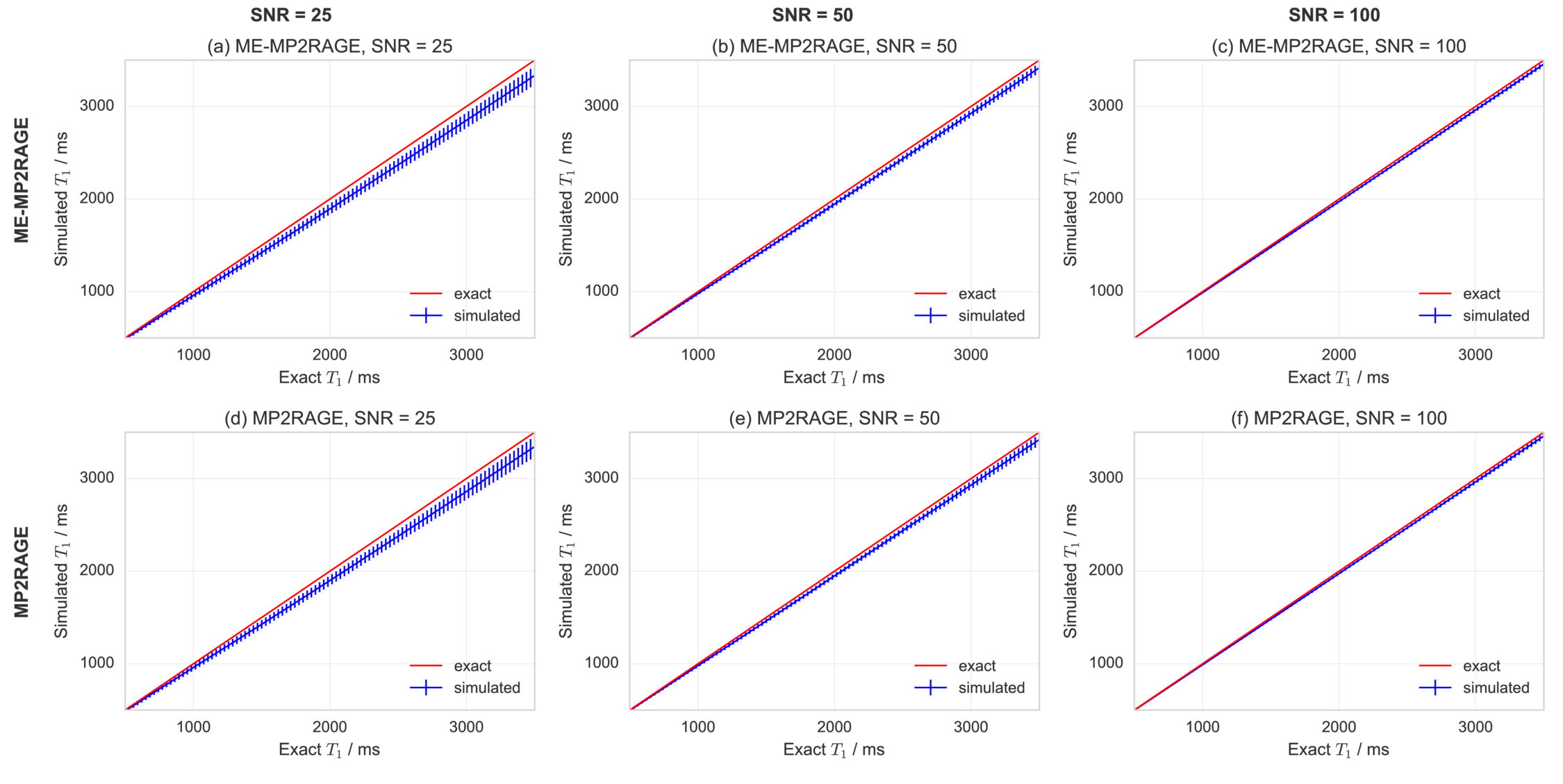

Supplement: S1 Fig — Plot of T1 estimates obtained with simulations versus the exact input values (range 0.5–3.5 s) as a function of the SNR. Error bars indicate the standard deviations, σT, for N = 20000 simulations. Different columns correspond to different SNR levels of (a,d) 25, (b,e) 50, and (c,f) 100, while the two rows show the results for (a–c) ME-MP2RAGE with TI,(1,2) = 800, 2400 ms and for (d–f) MP2RAGE with TI,(1,2) = 750, 2900 ms. (TIFF) [file pone.0169265.s003.tiff]

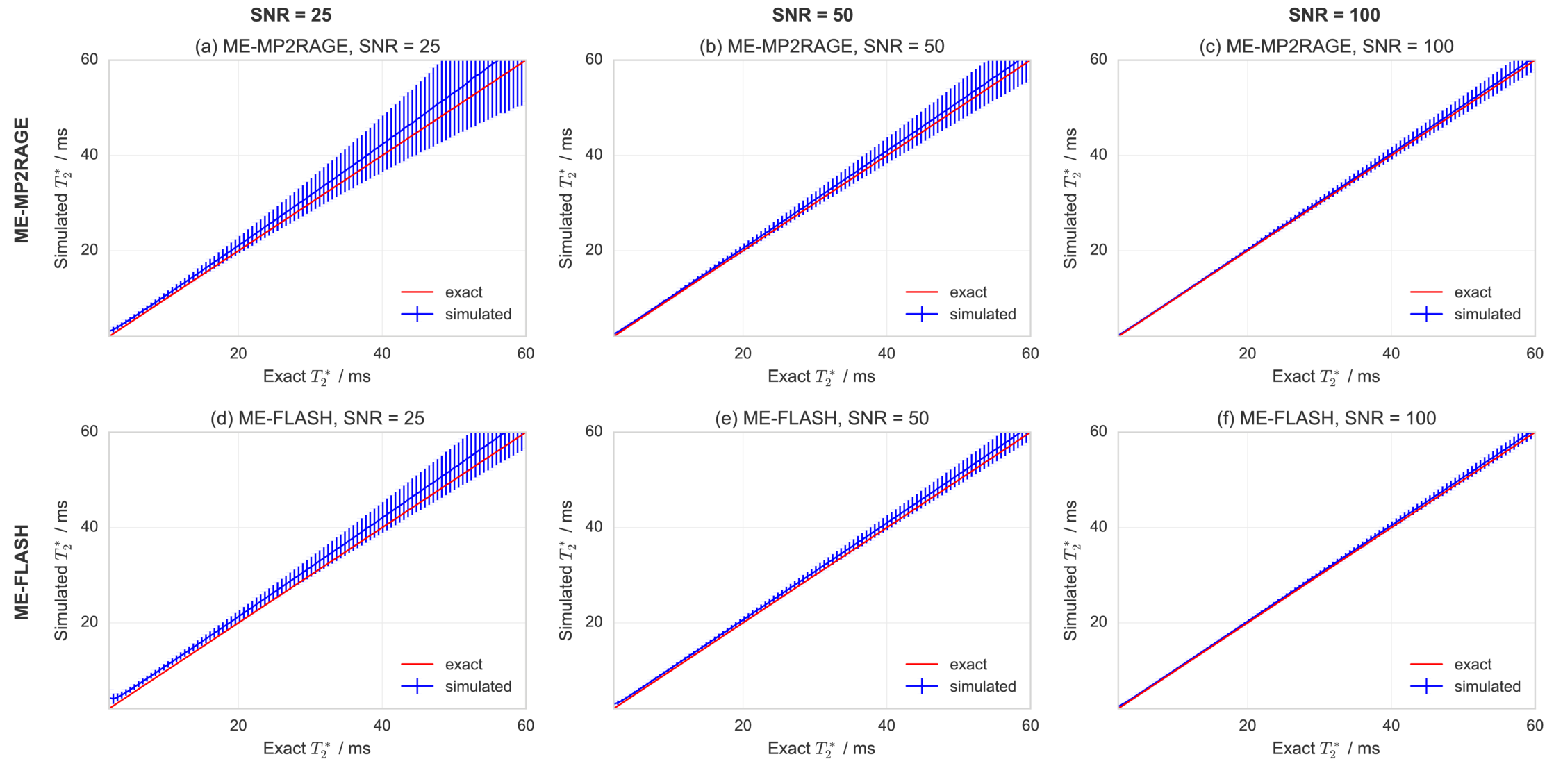

Supplement: S2 Fig — Plot of T2* estimates obtained with simulations versus the exact input values (range 2–60 ms) as a function of the SNR. Error bars indicate the standard deviations, σT, for N = 20000 simulations. Different columns correspond to different SNR levels of (a,d) 25, (b,e) 50, and (c,f) 100, while the two rows show the results for (a–c) ME-MP2RAGE with nE = 4, TE,1 = 2.5 ms, and ΔTE ≈ 4.2 ms and for (d–f) ME-FLASH with nE = 5, TE,1 = 3.0 ms, and ΔTE = 6.0 ms. (TIFF) [file pone.0169265.s004.tiff]

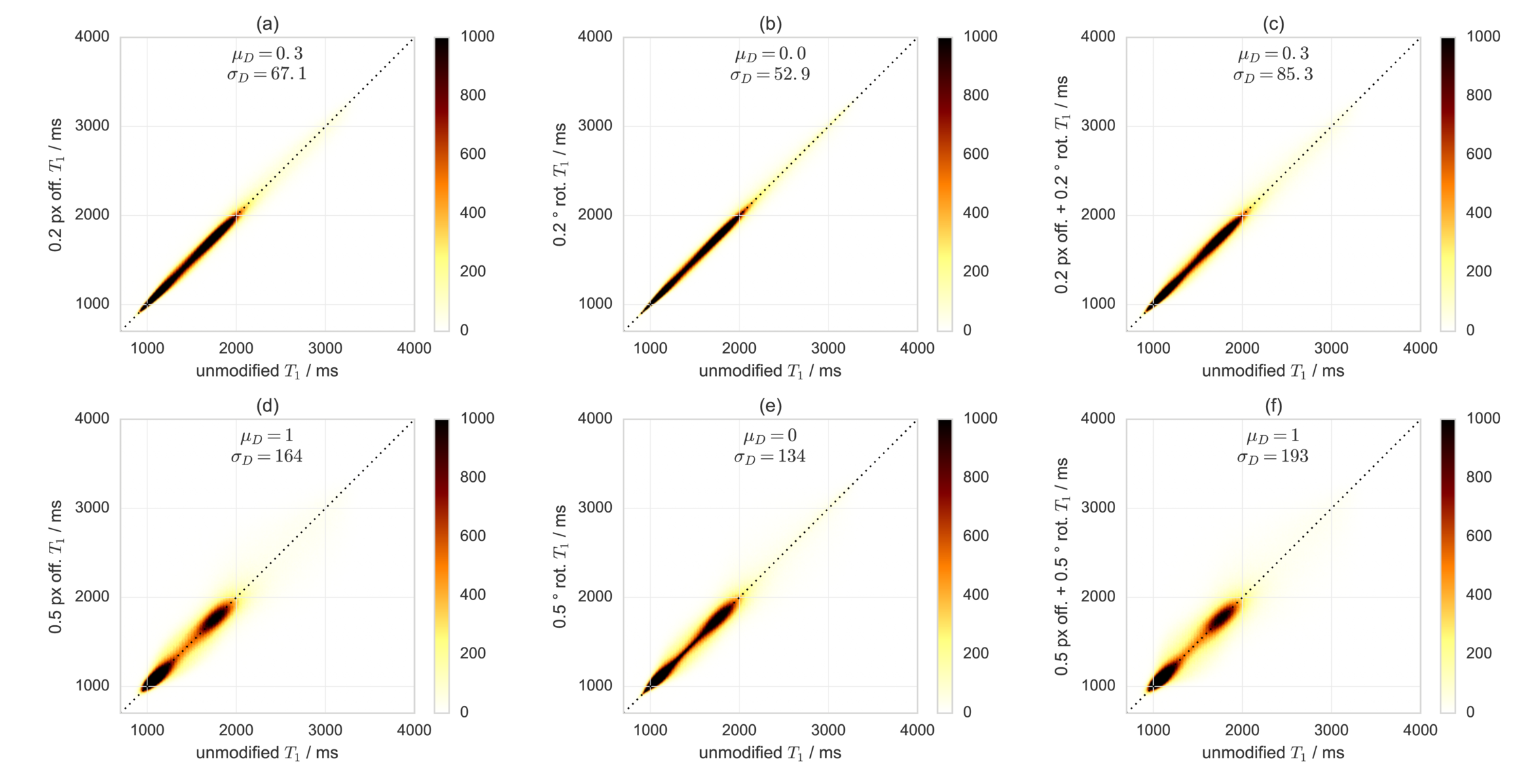

Supplement: S3 Fig — The x-axis refers to the unmodified map, while the y-axis refers to the same map after application of: (a) a translation by 0.2 px, (b) a rotation by 0.2°, (c) a rotation by 0.2° followed by a translation by 0.2 px, (d) a translation by 0.5 px, (e) a rotation by 0.5°, (f) a rotation by 0.5° followed by a translation by 0.5 px. More details can be found in S11 Table. (TIFF) [file pone.0169265.s005.tiff]

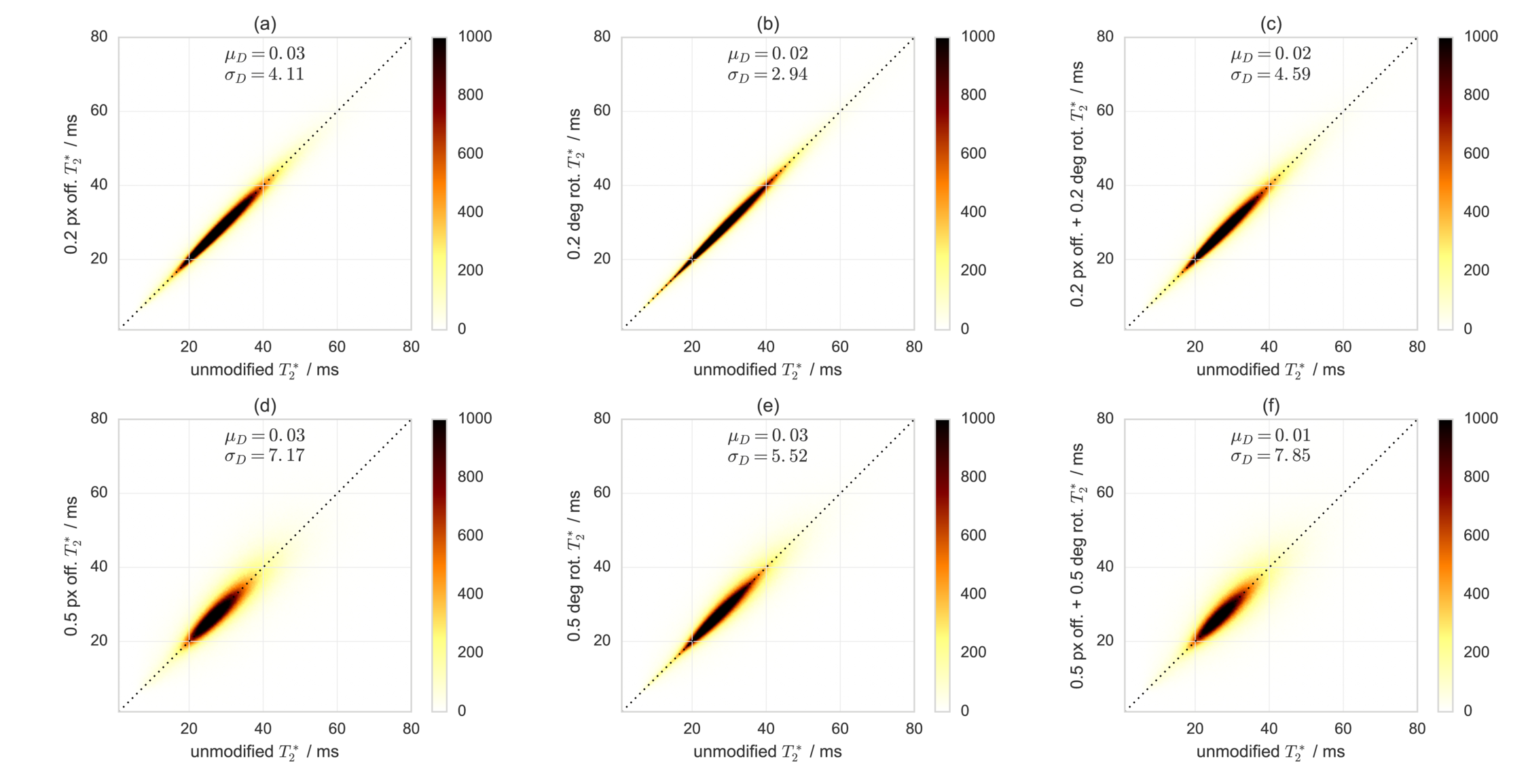

Supplement: S4 Fig — The x-axis refers to the unmodified map, while the y-axis refers to the same map after application of: (a) a translation by 0.2 px, (b) a rotation by 0.2°, (c) a rotation by 0.2° followed by a translation by 0.2 px, (d) a translation by 0.5 px, (e) a rotation by 0.5°, (f) a rotation by 0.5° followed by a translation by 0.5 px. More details can be found in S12 Table. (TIFF) [file pone.0169265.s006.tiff]

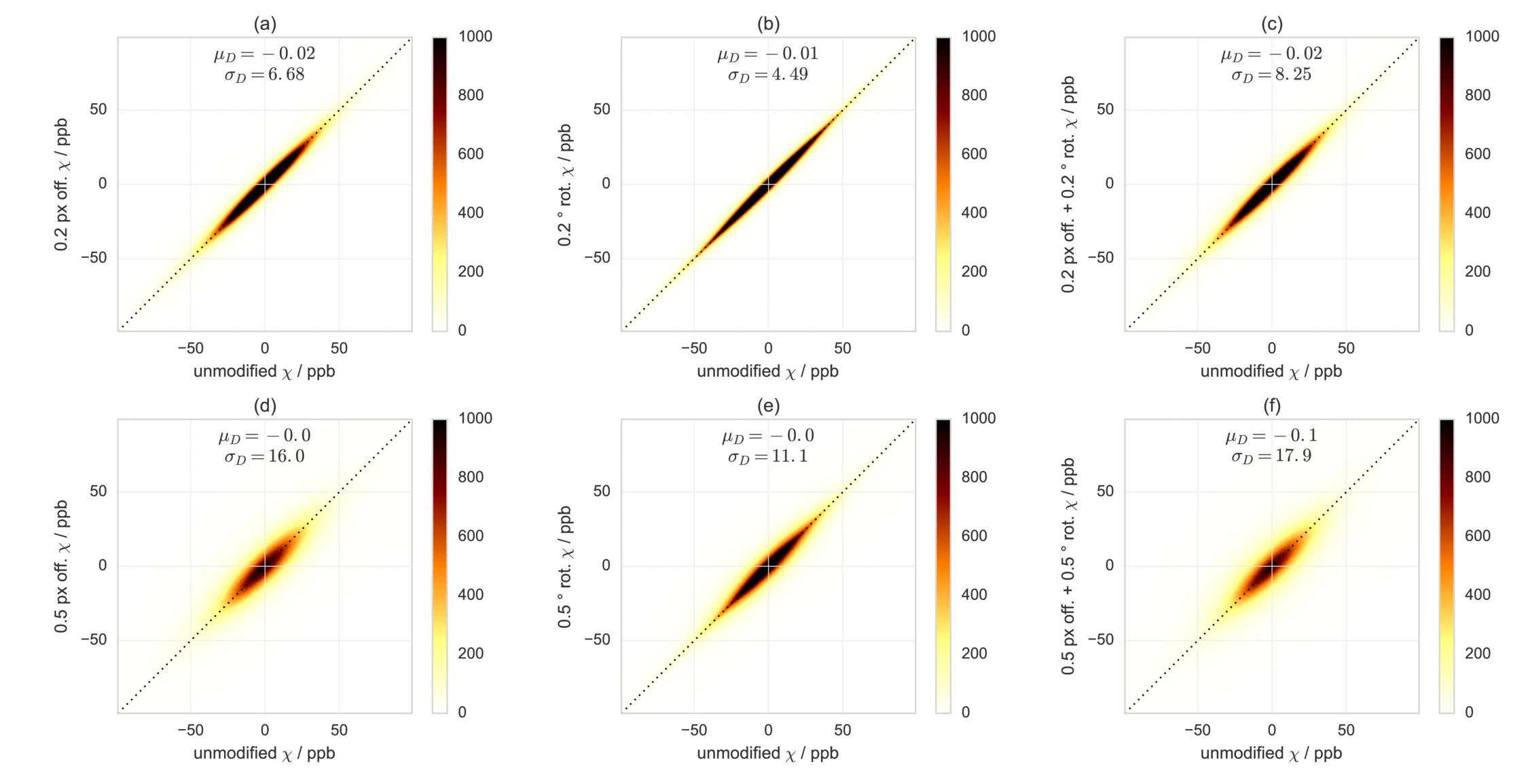

Supplement: S5 Fig — The x-axis refers to the unmodified map, while the y-axis refers to the same map after application of: (a) a translation by 0.2 px, (b) a rotation by 0.2°, (c) a rotation by 0.2° followed by a translation by 0.2 px, (d) a translation by 0.5 px, (e) a rotation by 0.5°, (f) a rotation by 0.5° followed by a translation by 0.5 px. More details can be found in S13 Table. (TIFF) [file pone.0169265.s007.tiff]

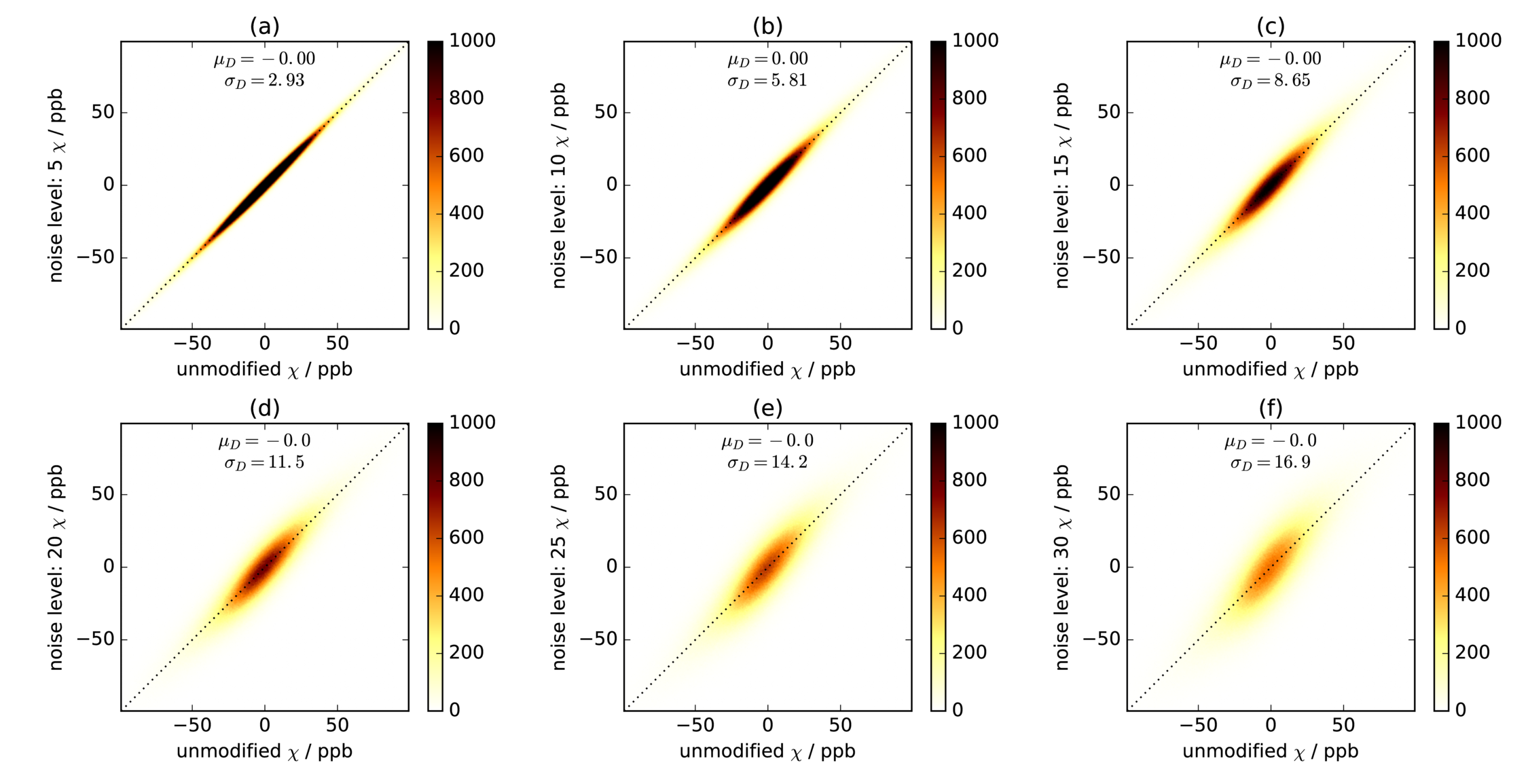

Supplement: S6 Fig — The x-axis refers to the unmodified map, while the y-axis refers to a map calculated from the same source complex image after adding Gaussian noise with a SD of (a) 5, (b) 10, (c) 15, (d) 20, (e) 25, (f) 30 arbitrary intensity units, corresponding to up to 100% increase in SNR. The original SNR was estimated as the maximum of the magnitude image divided by the standard deviation of the real image in a region where no signal was expected. More details can be found in S14 Table. (TIFF) [file pone.0169265.s008.tiff]

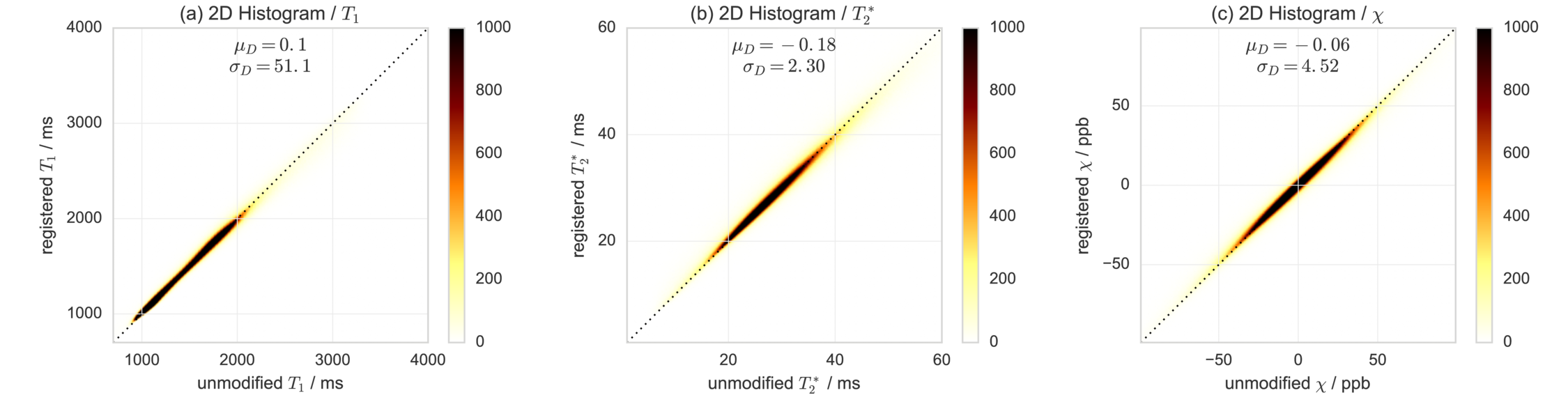

Supplement: S7 Fig — Results for (a) T1, (b) T2* and (c) χ maps are shown. The x-axis refers to the unmodified map, while the y-axis refers to the self-registered map, i.e. the same map after the application of a 10° rotation and then registered back (using FLIRT) onto the original map. (TIFF) [file pone.0169265.s009.tiff]

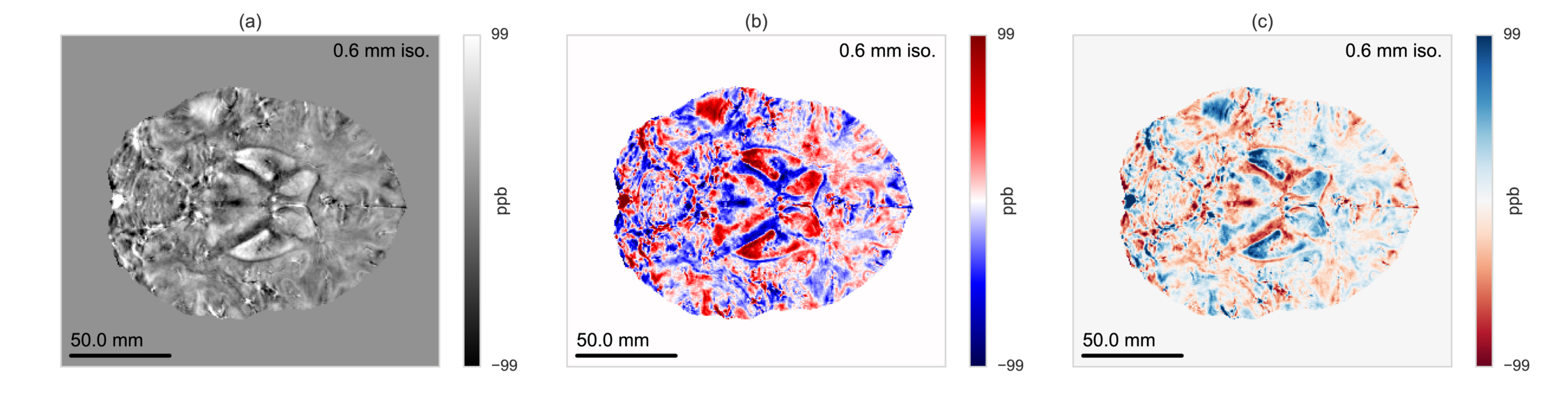

Supplement: S8 Fig — The same magnetic susceptibility map is displayed with the standard gray scale color map (a), a diverging color map with non-linear (b) and (almost) linear luminance (c). The divergin color map with linear luminance (see also: http://matplotlib.org/users/colormaps.html and “A Better Default Colormap for Matplotlib.” Berkeley Institute for Data Science, July 17, 2015. https://bids.berkeley.edu/resources/videos/better-default-colormap-matplotlib) may be a better option (although not used in previous literature) because it allows for a clear separation of positive and negative values, while retaining as much quantitative information as a gray color scale. This is actually interesting in view of the applications where a separation from paramagnetic and diamagnetic substances is desired. On the other hand, the arbitrary reference currently limits its efficacy and its adoption could be postponed until the community reaches a consensus on this. (TIFF) [file pone.0169265.s010.tiff]

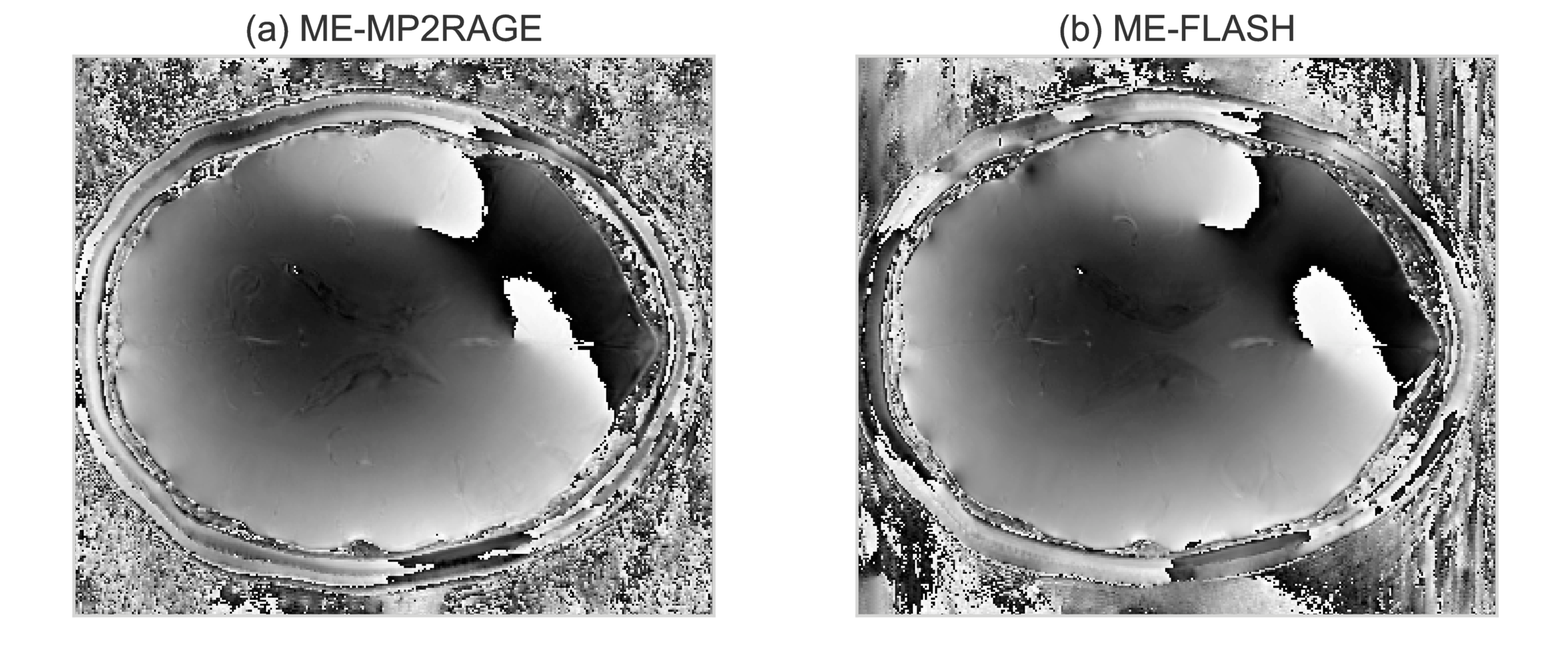

Supplement: S9 Fig — Phase images from the same subject and session are shown side by side for the ME-MP2RAGE acquisition and the ME-FLASH acquisition. The position of the pole artifact is consistent between the two acquisition (within the limits of subject’s motion), thus suggesting a limited impact on test-retest reproducibility. (TIFF) [file pone.0169265.s011.tiff]
