# Supplementary material for: Simultaneous Quantitative MRI Mapping of T1, T2* and Magnetic Susceptibility with Multi-Echo MP2RAGE
Source: PLoS One. 2017 Jan 12;12(1):e0169265. doi: 10.1371/journal.pone.0169265 (PMC5230783; doi:10.1371/journal.pone.0169265)
Supplement: S3 Table — Average and standard deviation of σT values in simulations of T1 mapping (assumed T1 values between 0.5 and 3.5 s) to evaluate the SNR influence. (PDF) [file pone.0169265.s012.pdf]

SNR level [#]	$T_{I,1}$ [ms]	$T_{I,2}$ [ms]	μ_σ [ms]	σ_σ [ms]
25	800	2000	62.8	30.3
25	1000	2000	66.2	28.1
25	800	2400	58.2	23.5
25	1000	2400	59.8	20.0
25	1200	2400	63.9	15.4
25	1400	2400	72.8	9.96
25	800	2800	57.7	21.6
25	1000	2800	58.4	18.5
25	1200	2800	61.5	14.8
25	1400	2800	67.8	10.6
25	800	3200	58.4	21.9
25	1000	3200	58.6	19.2
25	1200	3200	61.1	16.2
25	1400	3200	66.2	13.2
25	800	3600	59.8	22.8
25	1000	3600	59.6	20.5
25	1200	3600	61.5	18.0
25	1400	3600	65.6	15.4
50	800	2000	32.4	16.2
50	1000	2000	34.2	15.5
50	800	2400	30.0	12.5
50	1000	2400	30.7	10.9
50	1200	2400	32.6	8.55
50	1400	2400	36.7	5.90
50	800	2800	29.5	11.3
50	1000	2800	29.8	9.70
50	1200	2800	31.2	7.70
50	1400	2800	34.1	5.41
50	800	3200	29.8	11.1
50	1000	3200	29.9	9.75
50	1200	3200	31.0	8.20
50	1400	3200	33.3	6.51
50	800	3600	30.6	11.5
50	1000	3600	30.4	10.3
50	1200	3600	31.2	8.96
50	1400	3600	33.1	7.58
100	800	2000	16.5	8.41
100	1000	2000	17.4	8.14
100	800	2400	15.2	6.41
100	1000	2400	15.5	5.64
100	1200	2400	16.5	4.58
100	1400	2400	18.5	3.24
100	800	2800	14.9	5.73
100	1000	2800	15.1	4.97
100	1200	2800	15.7	3.94
100	1400	2800	17.1	2.78
100	800	3200	15.1	5.63
100	1000	3200	15.1	4.93
100	1200	3200	15.6	4.13
100	1400	3200	16.7	3.24
100	800	3600	15.5	5.82
100	1000	3600	15.3	5.18
100	1200	3600	15.7	4.47
100	1400	3600	16.6	3.76
