# Supplementary material for: Simultaneous Quantitative MRI Mapping of T1, T2* and Magnetic Susceptibility with Multi-Echo MP2RAGE
Source: PLoS One. 2017 Jan 12;12(1):e0169265. doi: 10.1371/journal.pone.0169265 (PMC5230783; doi:10.1371/journal.pone.0169265)
Supplement: S4 Table — Average and standard deviation of σT values in simulations of T2* mapping (assumed T2* values between 2 and 60 ms) to evaluate the SNR influence. (PDF) [file pone.0169265.s013.pdf]

SNR level [#]	T_E range [ms]	n_E [#]	ΔT_E [ms]	μ_σ [ms]	σ_σ [ms]
25	2.0–15.0	3	4.3	4.86	3.94
25	2.0–20.0	3	6.0	3.61	2.67
25	2.0–30.0	3	9.3	2.70	1.67
25	3.0–15.0	3	4.0	5.53	4.55
25	3.0–20.0	3	5.7	3.92	2.87
25	3.0–30.0	3	9.0	2.88	1.74
25	2.0–15.0	4	3.2	4.49	3.63
25	2.0–20.0	4	4.5	3.35	2.51
25	2.0–30.0	4	7.0	2.47	1.61
25	3.0–15.0	4	3.0	5.07	4.12
25	3.0–20.0	4	4.2	3.64	2.69
25	3.0–30.0	4	6.8	2.63	1.66
25	2.0–15.0	5	2.6	4.16	3.35
25	2.0–20.0	5	3.6	3.12	2.35
25	2.0–30.0	5	5.6	2.29	1.52
25	3.0–15.0	5	2.4	4.67	3.77
25	3.0–20.0	5	3.4	3.38	2.51
25	3.0–30.0	5	5.4	2.44	1.57
50	2.0–15.0	3	4.3	2.17	1.67
50	2.0–20.0	3	6.0	1.69	1.21
50	2.0–30.0	3	9.3	1.30	0.789
50	3.0–15.0	3	4.0	2.40	1.84
50	3.0–20.0	3	5.7	1.82	1.29
50	3.0–30.0	3	9.0	1.38	0.816
50	2.0–15.0	4	3.2	2.04	1.58
50	2.0–20.0	4	4.5	1.57	1.16
50	2.0–30.0	4	7.0	1.19	0.766
50	3.0–15.0	4	3.0	2.25	1.74
50	3.0–20.0	4	4.2	1.70	1.23
50	3.0–30.0	4	6.8	1.26	0.792
50	2.0–15.0	5	2.6	1.91	1.49
50	2.0–20.0	5	3.6	1.47	1.09
50	2.0–30.0	5	5.6	1.10	0.727
50	3.0–15.0	5	2.4	2.10	1.63
50	3.0–20.0	5	3.4	1.59	1.16
50	3.0–30.0	5	5.4	1.17	0.751
100	2.0–15.0	3	4.3	1.05	0.806
100	2.0–20.0	3	6.0	0.826	0.590
100	2.0–30.0	3	9.3	0.644	0.385
100	3.0–15.0	3	4.0	1.16	0.878
100	3.0–20.0	3	5.7	0.889	0.628
100	3.0–30.0	3	9.0	0.682	0.400
100	2.0–15.0	4	3.2	0.991	0.766
100	2.0–20.0	4	4.5	0.771	0.565
100	2.0–30.0	4	7.0	0.585	0.376
100	3.0–15.0	4	3.0	1.09	0.837
100	3.0–20.0	4	4.2	0.830	0.601
100	3.0–30.0	4	6.8	0.619	0.388
100	2.0–15.0	5	2.6	0.931	0.724
100	2.0–20.0	5	3.6	0.723	0.535
100	2.0–30.0	5	5.6	0.543	0.359
100	3.0–15.0	5	2.4	1.02	0.787
100	3.0–20.0	5	3.4	0.778	0.567
100	3.0–30.0	5	5.4	0.573	0.369
