# Supplementary material for: Simultaneous Quantitative MRI Mapping of T1, T2* and Magnetic Susceptibility with Multi-Echo MP2RAGE
Source: PLoS One. 2017 Jan 12;12(1):e0169265. doi: 10.1371/journal.pone.0169265 (PMC5230783; doi:10.1371/journal.pone.0169265)
Supplement: S5 Table — The order of the listed acquisition parameters is: nominal isotropic resolution, TR,seq, α1,2, TI,(1,2), and TE. (PDF) [file pone.0169265.s014.pdf]

Test	Ref.	μ_D [ms]	σ_D [ms]	$\mu_{\|D\|}$ [ms]	$\sigma_{\|D\|}$ [ms]	r^2 [#]
ME-MP2RAGE / 0.9 mm / 8000 ms / $5°, 10°$ / $900, 2750$ ms / 2.65 ms	MP2RAGE / 0.9 mm / 5000 ms / $5°, 3°$ / $900, 2750$ ms / 2.95 ms	-47.3	158	95.0	135	0.898
ME-MP2RAGE / 0.9 mm / 5000 ms / $5°, 3°$ / $900, 2750$ ms / 2.65 ms	MP2RAGE / 0.9 mm / 5000 ms / $5°, 3°$ / $900, 2750$ ms / 2.95 ms	-39.0	77.6	62.7	60.1	0.969
MP2RAGE / 0.9 mm / 5000 ms / $5°, 3°$ / $900, 2750$ ms / 2.95 ms	MP2RAGE / 0.9 mm / 8000 ms / $4°, 5°$ / $900, 2750$ ms / 2.95 ms	-9.63	97.2	57.1	79.3	0.953
MP2RAGE / 0.9 mm / 5000 ms / $5°, 3°$ / $900, 2750$ ms / 2.95 ms	MP2RAGE / 0.9 mm / 5000 ms / $5°, 3°$ / $900, 2750$ ms / 2.95 ms	0.528	60.5	39.3	46.1	0.981
ME-MP2RAGE / 0.9 mm / 5000 ms / $5°, 3°$ / $900, 2750$ ms / 2.65 ms	ME-MP2RAGE / 0.9 mm / 8000 ms / $5°, 10°$ / $900, 2750$ ms / 2.65 ms	6.63	136	87.7	104	0.907
ME-MP2RAGE / 0.7 mm / 8000 ms / $4°, 9°$ / $1100, 3500$ ms / 2.32 ms	MP2RAGE / 0.7 mm / 8000 ms / $4°, 5°$ / $1000, 3300$ ms / 3.31 ms	-64.7	146	117	109	0.884
ME-MP2RAGE / 0.9 mm / 5000 ms / $7°, 7°$ / $900, 2750$ ms / 2.65 ms	MP2RAGE / 0.9 mm / 5000 ms / $5°, 3°$ / $900, 2750$ ms / 3.12 ms	-260	195	264	190	0.930
