# Supplementary material for: Simultaneous Quantitative MRI Mapping of T1, T2* and Magnetic Susceptibility with Multi-Echo MP2RAGE
Source: PLoS One. 2017 Jan 12;12(1):e0169265. doi: 10.1371/journal.pone.0169265 (PMC5230783; doi:10.1371/journal.pone.0169265)
Supplement: S6 Table — The order of the listed acquisition parameters is: nominal isotropic resolution, TR,seq, α1,2, TI,(1,2), and TE values for ME-MP2RAGE; nominal isotropic resolution, TR, α and TE values for ME-FLASH. (PDF) [file pone.0169265.s015.pdf]

Test	Ref.	μ_D [ms]	σ_D [ms]	$\mu_{\|D\|}$ [ms]	$\sigma_{\|D\|}$ [ms]	r^2 [#]
ME-MP2RAGE / 0.9 mm / 7000 ms / $5°, 10°$ / $900, 2750$ ms / 2.65, 5.33, 8.01, 10.97 ms	ME-FLASH / 0.9 mm / 32 ms / $10°$ / 4.08, 10.20, 16.32, 22.40 ms	0.439	7.87	4.84	6.23	0.416
ME-MP2RAGE / 0.9 mm / 5000 ms / $5°, 3°$ / $900, 2750$ ms / 2.65, 5.33, 8.01, 10.97 ms	ME-FLASH / 0.9 mm / 32 ms / $10°$ / 4.08, 10.20, 16.32, 22.40 ms	−0.255	8.54	5.34	6.67	0.373
ME-MP2RAGE / 0.9 mm / 8000 ms / $5°, 10°$ / $900, 2750$ ms / 2.65, 5.33, 8.01, 10.97 ms	ME-FLASH / 0.9 mm / 35 ms / $12°$ / 7.14, 13.26, 19.37, 25.47 ms	0.514	8.43	5.36	6.53	0.302
ME-FLASH / 0.9 mm / 35 ms / $12°$ / 7.14, 13.26, 19.37, 25.47 ms	ME-FLASH / 0.9 mm / 35 ms / $12°$ / 7.14, 13.26, 19.37, 25.47 ms	−0.0244	5.80	3.65	4.51	0.595
ME-MP2RAGE / 0.9 mm / 8000 ms / $5°, 10°$ / $900, 2750$ ms / 2.65, 5.33, 8.01, 10.97 ms	ME-MP2RAGE / 0.9 mm / 8000 ms / $5°, 10°$ / $900, 2750$ ms / 2.65, 5.33, 8.01, 10.97 ms	0.209	7.61	4.66	6.02	0.549
ME-MP2RAGE / 0.9 mm / 8000 ms / $5°, 10°$ / $900, 2750$ ms / 2.65, 5.33, 8.01, 10.97 ms	ME-MP2RAGE / 0.9 mm / 7000 ms / $5°, 10°$ / $900, 2750$ ms / 2.65, 5.33, 8.01, 10.97 ms	0.399	7.12	4.43	5.58	0.551
ME-MP2RAGE / 0.9 mm / 8000 ms / $5°, 10°$ / $900, 2750$ ms / 2.65, 5.33, 8.01, 10.97 ms	ME-MP2RAGE / 0.9 mm / 5000 ms / $5°, 3°$ / $900, 2750$ ms / 2.65, 5.33, 8.01, 10.97 ms	1.12	7.98	5.12	6.23	0.477
