# Supplementary material for: Simultaneous Quantitative MRI Mapping of T1, T2* and Magnetic Susceptibility with Multi-Echo MP2RAGE
Source: PLoS One. 2017 Jan 12;12(1):e0169265. doi: 10.1371/journal.pone.0169265 (PMC5230783; doi:10.1371/journal.pone.0169265)
Supplement: S7 Table — The order of the listed acquisition parameters is: nominal isotropic resolution, TR,seq, α1,2, TI,(1,2), and TE for ME-MP2RAGE; nominal isotropic resolution, TR, α and TE for ME-FLASH. (PDF) [file pone.0169265.s016.pdf]

Test	Ref.	μ_D [ppb]	σ_D [ppb]	$\mu_{\|D\|}$ [ppb]	$\sigma_{\|D\|}$ [ppb]	r^2 [#]
ME-MP2RAGE / 0.9 mm / 8000 ms / $5°, 10°$ / $900, 2750$ ms / 10.97 ms	ME-FLASH / 0.9 mm / 32 ms / 10° / 10.20 ms	−0.369	17.9	12.2	13.1	0.630
ME-MP2RAGE / 0.9 mm / 5000 ms / $5°, 3°$ / $900, 2750$ ms / 10.97 ms	ME-FLASH / 0.9 mm / 32 ms / 10° / 10.20 ms	−0.319	16.8	11.7	12.2	0.679
ME-MP2RAGE / 0.9 mm / 5000 ms / $5°, 3°$ / $900, 2750$ ms / 10.97 ms	ME-FLASH / 0.9 mm / 32 ms / 10° / 16.32 ms	−0.180	17.2	12.2	12.1	0.676
ME-FLASH / 0.9 mm / 32 ms / 10° / 10.20 ms	ME-FLASH / 0.9 mm / 32 ms / 10° / 10.20 ms	0.210	12.9	9.24	9.07	0.816
ME-MP2RAGE / 0.9 mm / 8000 ms / $5°, 10°$ / $900, 2750$ ms / 10.97 ms	ME-MP2RAGE / 0.9 mm / 5000 ms / $5°, 3°$ / $900, 2750$ ms / 10.97 ms	−0.0311	13.4	9.53	9.45	0.788
ME-MP2RAGE / 0.9 mm / 8000 ms / $5°, 10°$ / $900, 2750$ ms / 10.97 ms	ME-MP2RAGE / 0.9 mm / 8000 ms / $5°, 10°$ / $900, 2750$ ms / 10.97 ms	0.0771	12.7	8.61	9.39	0.828
