# Supplementary material for: Simultaneous Quantitative MRI Mapping of T1, T2* and Magnetic Susceptibility with Multi-Echo MP2RAGE
Source: PLoS One. 2017 Jan 12;12(1):e0169265. doi: 10.1371/journal.pone.0169265 (PMC5230783; doi:10.1371/journal.pone.0169265)
Supplement: S8 Table — Variations of the correlation coefficients, and means and SDs of image volume differences (as defined in Eqs 4 and 5) obtained in Study 2 for T1 maps across different subjects with the acquisition parameters from Table 1. The last part of the table are the group averages μg and SDs σg according to the acquisition scheme. (PDF) [file pone.0169265.s017.pdf]

Subj.	Test	Ref.	μ_D [ms]	σ_D [ms]	$\mu_{\|D\|}$ [ms]	$\sigma_{\|D\|}$ [ms]	r^2 [#]
s01	ME-MP2RAGE	MP2RAGE	11.1	130	88.2	95.6	0.886
s02	ME-MP2RAGE	MP2RAGE	13.6	144	95.0	109	0.871
s02	ME-MP2RAGE	MP2RAGE	18.8	127	83.5	98.2	0.898
s02	ME-MP2RAGE	MP2RAGE	9.24	157	104	118	0.849
s02	ME-MP2RAGE	MP2RAGE	14.6	140	92.1	106	0.880
s03	ME-MP2RAGE	MP2RAGE	5.29	142	95.6	105	0.868
s03	ME-MP2RAGE	MP2RAGE	11.1	182	126	132	0.787
s11	ME-MP2RAGE	MP2RAGE	22.6	133	95.0	96.1	0.883
s12	ME-MP2RAGE	MP2RAGE	5.71	164	108	123	0.828
s12	ME-MP2RAGE	MP2RAGE	11.1	137	89.2	105	0.878
s12	ME-MP2RAGE	MP2RAGE	7.95	152	103	112	0.846
s12	ME-MP2RAGE	MP2RAGE	12.7	125	84.9	92.5	0.896
s13	ME-MP2RAGE	MP2RAGE	6.04	132	89.2	97.6	0.891
s13	ME-MP2RAGE	MP2RAGE	7.90	135	91.3	100	0.887
s18	ME-MP2RAGE	MP2RAGE	23.4	140	95.6	104	0.878
s18	ME-MP2RAGE	MP2RAGE	12.6	137	92.9	102	0.881
s18	ME-MP2RAGE	MP2RAGE	15.5	126	84.3	94.8	0.901
s18	ME-MP2RAGE	MP2RAGE	4.86	127	84.0	95.9	0.898
s02	MP2RAGE	MP2RAGE	5.34	98.3	68.9	70.4	0.945
s12	MP2RAGE	MP2RAGE	6.30	122	82.0	90.3	0.912
s18	MP2RAGE	MP2RAGE	−10.3	91.7	62.3	68.0	0.955
s02	ME-MP2RAGE	ME-MP2RAGE	3.81	110	72.3	82.7	0.922
s03	ME-MP2RAGE	ME-MP2RAGE	−5.14	155	102	117	0.840
s12	ME-MP2RAGE	ME-MP2RAGE	−3.14	119	77.5	89.9	0.903
s13	ME-MP2RAGE	ME-MP2RAGE	−2.02	75.5	52.6	54.2	0.964
s15	ME-MP2RAGE	ME-MP2RAGE	5.20	103	67.7	78.4	0.923
s18	ME-MP2RAGE	ME-MP2RAGE	7.35	86.9	63.1	61.0	0.952
μ_g	ME-MP2RAGE	MP2RAGE	11.9	141	94.6	105	0.873
σ_g	ME-MP2RAGE	MP2RAGE	5.40	14.5	10.2	10.4	0.028
μ_g	MP2RAGE	MP2RAGE	0.461	104	71.1	76.2	0.937
σ_g	MP2RAGE	MP2RAGE	7.59	12.9	8.20	9.98	0.018
μ_g	ME-MP2RAGE	ME-MP2RAGE	1.01	108	72.4	80.5	0.917
σ_g	ME-MP2RAGE	ME-MP2RAGE	4.65	25.3	15.2	20.4	0.040
