# Supplementary material for: Simultaneous Quantitative MRI Mapping of T1, T2* and Magnetic Susceptibility with Multi-Echo MP2RAGE
Source: PLoS One. 2017 Jan 12;12(1):e0169265. doi: 10.1371/journal.pone.0169265 (PMC5230783; doi:10.1371/journal.pone.0169265)
Supplement: S9 Table — Variations of the correlation coefficients, and means and SDs of image volume differences (as defined in Eqs 4 and 5) obtained in Study 2 for T2* maps across different subjects with the acquisition parameters from Table 1. The last part of the table are the group averages μg and SDs σg according to the acquisition scheme. (PDF) [file pone.0169265.s018.pdf]

Subj.	Test	Ref.	μ_D [ms]	σ_D [ms]	$\mu_{\|D\|}$ [ms]	$\sigma_{\|D\|}$ [ms]	r^2 [#]
s02	ME-MP2RAGE	ME-FLASH	0.476	10.1	6.95	7.29	0.301
s02	ME-MP2RAGE	ME-FLASH	0.0590	10.0	6.93	7.24	0.300
s02	ME-MP2RAGE	ME-FLASH	0.171	10.8	7.44	7.87	0.253
s02	ME-MP2RAGE	ME-FLASH	−0.237	10.7	7.37	7.76	0.261
s03	ME-MP2RAGE	ME-FLASH	0.745	9.09	6.23	6.66	0.335
s03	ME-MP2RAGE	ME-FLASH	0.642	9.27	6.26	6.87	0.324
s03	ME-MP2RAGE	ME-FLASH	0.876	12.2	8.50	8.75	0.130
s03	ME-MP2RAGE	ME-FLASH	0.750	12.4	8.55	9.00	0.120
s11	ME-MP2RAGE	ME-FLASH	0.584	9.21	6.33	6.71	0.334
s11	ME-MP2RAGE	ME-FLASH	−1.74	11.4	7.86	8.43	0.237
s11	ME-MP2RAGE	ME-FLASH	−1.43	11.7	8.10	8.56	0.226
s12	ME-MP2RAGE	ME-FLASH	0.121	11.4	7.66	8.46	0.191
s12	ME-MP2RAGE	ME-FLASH	0.588	11.5	7.63	8.59	0.187
s12	ME-MP2RAGE	ME-FLASH	−0.207	10.4	6.87	7.85	0.283
s12	ME-MP2RAGE	ME-FLASH	0.281	10.3	6.65	7.81	0.301
s13	ME-MP2RAGE	ME-FLASH	0.402	8.67	5.64	6.60	0.390
s13	ME-MP2RAGE	ME-FLASH	0.500	8.89	5.71	6.83	0.358
s13	ME-MP2RAGE	ME-FLASH	0.0354	8.91	5.83	6.74	0.376
s13	ME-MP2RAGE	ME-FLASH	0.124	9.01	5.81	6.89	0.358
s14	ME-MP2RAGE	ME-FLASH	1.02	8.94	6.26	6.46	0.361
s14	ME-MP2RAGE	ME-FLASH	1.04	9.12	6.33	6.65	0.350
s14	ME-MP2RAGE	ME-FLASH	0.590	8.72	5.81	6.53	0.366
s14	ME-MP2RAGE	ME-FLASH	0.614	8.87	5.85	6.70	0.359
s18	ME-MP2RAGE	ME-FLASH	0.783	7.47	4.71	5.85	0.492
s18	ME-MP2RAGE	ME-FLASH	0.930	7.65	4.86	5.99	0.480
s19	ME-MP2RAGE	ME-FLASH	0.365	10.3	7.09	7.54	0.262
s19	ME-MP2RAGE	ME-FLASH	0.0863	8.93	6.12	6.50	0.364
s19	ME-MP2RAGE	ME-FLASH	0.487	8.83	5.95	6.54	0.347
s02	ME-FLASH	ME-FLASH	−0.402	8.81	6.35	6.12	0.374
s03	ME-FLASH	ME-FLASH	−0.0924	7.52	5.18	5.44	0.448
s12	ME-FLASH	ME-FLASH	0.509	8.15	5.56	5.98	0.457
s13	ME-FLASH	ME-FLASH	0.0898	7.68	5.16	5.69	0.483
s14	ME-FLASH	ME-FLASH	0.0112	7.28	4.93	5.35	0.497
s18	ME-FLASH	ME-FLASH	0.150	5.10	3.28	3.90	0.740
s02	ME-MP2RAGE	ME-MP2RAGE	0.312	10.7	7.15	7.90	0.301
s03	ME-MP2RAGE	ME-MP2RAGE	−0.0427	12.3	8.29	9.09	0.159
s11	ME-MP2RAGE	ME-MP2RAGE	2.40	11.6	8.08	8.70	0.247
s11	ME-MP2RAGE	ME-MP2RAGE	2.08	12.0	8.38	8.91	0.225
s11	ME-MP2RAGE	ME-MP2RAGE	−0.319	12.9	8.90	9.34	0.220
s12	ME-MP2RAGE	ME-MP2RAGE	0.297	12.4	8.13	9.42	0.160
s13	ME-MP2RAGE	ME-MP2RAGE	0.377	7.68	4.62	6.15	0.511
s14	ME-MP2RAGE	ME-MP2RAGE	0.442	8.91	6.02	6.58	0.394
s15	ME-MP2RAGE	ME-MP2RAGE	0.383	7.34	4.45	5.84	0.502
s19	ME-MP2RAGE	ME-MP2RAGE	0.206	9.71	6.55	7.17	0.366
s19	ME-MP2RAGE	ME-MP2RAGE	−0.236	8.79	5.88	6.54	0.442
s19	ME-MP2RAGE	ME-MP2RAGE	−0.397	8.68	5.79	6.48	0.422
μ_g	ME-MP2RAGE	ME-FLASH	0.309	9.81	6.62	7.27	0.309
σ_g	ME-MP2RAGE	ME-FLASH	0.625	1.29	0.985	0.872	0.087
μ_g	ME-FLASH	ME-FLASH	0.0443	7.42	5.08	5.42	0.500
σ_g	ME-FLASH	ME-FLASH	0.273	1.15	0.923	0.729	0.114
μ_g	ME-MP2RAGE	ME-MP2RAGE	0.459	10.3	6.85	7.68	0.329
σ_g	ME-MP2RAGE	ME-MP2RAGE	0.846	1.89	1.45	1.30	0.122
