# Supplementary material for: Simultaneous Quantitative MRI Mapping of T1, T2* and Magnetic Susceptibility with Multi-Echo MP2RAGE
Source: PLoS One. 2017 Jan 12;12(1):e0169265. doi: 10.1371/journal.pone.0169265 (PMC5230783; doi:10.1371/journal.pone.0169265)
Supplement: S10 Table — Variations of the correlation coefficients, and means and SDs of image volume differences (as defined in Eqs 4 and 5) obtained in Study 2 for χ maps across different subjects with the acquisition parameters from Table 1. The last part of the table are the group averages μg and SDs σg according to the acquisition scheme. (PDF) [file pone.0169265.s019.pdf]

Subj.	Test	Ref.	μ_D [ppb]	σ_D [ppb]	$\mu_{\lvert D\rvert}$ [ppb]	$\sigma_{\lvert D\rvert}$ [ppb]	r^2 [#]
s02	ME-MP2RAGE	ME-FLASH	−0.235	20.7	14.9	14.3	0.477
s02	ME-MP2RAGE	ME-FLASH	−0.506	23.0	16.6	15.9	0.389
s02	ME-MP2RAGE	ME-FLASH	−0.0839	19.3	13.9	13.4	0.502
s02	ME-MP2RAGE	ME-FLASH	−0.133	18.2	13.2	12.6	0.574
s02	ME-MP2RAGE	ME-FLASH	−0.405	20.7	14.9	14.3	0.482
s02	ME-MP2RAGE	ME-FLASH	−0.00818	16.8	12.0	11.8	0.604
s03	ME-MP2RAGE	ME-FLASH	−0.183	24.6	18.0	16.8	0.263
s03	ME-MP2RAGE	ME-FLASH	−0.0619	22.4	16.3	15.4	0.317
s03	ME-MP2RAGE	ME-FLASH	−0.118	17.0	12.4	11.7	0.606
s03	ME-MP2RAGE	ME-FLASH	−0.0152	15.0	10.7	10.6	0.657
s11	ME-MP2RAGE	ME-FLASH	−0.169	19.6	14.1	13.6	0.576
s12	ME-MP2RAGE	ME-FLASH	−0.431	21.9	15.8	15.2	0.453
s12	ME-MP2RAGE	ME-FLASH	−0.139	19.2	13.6	13.5	0.527
s12	ME-MP2RAGE	ME-FLASH	−0.203	22.6	16.5	15.4	0.439
s12	ME-MP2RAGE	ME-FLASH	0.0883	18.1	13.1	12.5	0.587
s13	ME-MP2RAGE	ME-FLASH	−0.168	16.9	12.0	11.9	0.661
s13	ME-MP2RAGE	ME-FLASH	−0.0403	13.9	9.87	9.88	0.745
s13	ME-MP2RAGE	ME-FLASH	−0.163	16.9	12.1	11.8	0.662
s13	ME-MP2RAGE	ME-FLASH	−0.0362	13.5	9.54	9.59	0.759
s14	ME-MP2RAGE	ME-FLASH	−0.174	16.4	11.7	11.6	0.639
s14	ME-MP2RAGE	ME-FLASH	0.0407	14.4	10.1	10.3	0.693
s18	ME-MP2RAGE	ME-FLASH	−0.137	14.1	10.1	9.92	0.741
s18	ME-MP2RAGE	ME-FLASH	−0.000602	12.6	9.04	8.86	0.778
s18	ME-MP2RAGE	ME-FLASH	−0.155	14.5	10.3	10.3	0.727
s18	ME-MP2RAGE	ME-FLASH	−0.0198	12.5	8.83	8.83	0.784
s19	ME-MP2RAGE	ME-FLASH	−0.247	21.4	15.3	14.9	0.458
s02	ME-FLASH	ME-FLASH	−0.250	14.8	10.6	10.3	0.725
s02	ME-FLASH	ME-FLASH	0.130	17.4	12.7	11.9	0.600
s02	ME-FLASH	ME-FLASH	0.395	20.0	14.5	13.8	0.502
s03	ME-FLASH	ME-FLASH	0.100	16.9	12.4	11.6	0.604
s12	ME-FLASH	ME-FLASH	0.285	18.3	13.3	12.6	0.607
s13	ME-FLASH	ME-FLASH	0.119	15.4	11.0	10.7	0.714
s14	ME-FLASH	ME-FLASH	0.215	15.0	10.7	10.5	0.691
s18	ME-FLASH	ME-FLASH	0.134	12.5	8.85	8.91	0.790
s02	ME-MP2RAGE	ME-MP2RAGE	−0.0751	18.8	13.2	13.5	0.536
s03	ME-MP2RAGE	ME-MP2RAGE	−0.0526	22.1	16.0	15.4	0.336
s12	ME-MP2RAGE	ME-MP2RAGE	−0.243	21.4	15.2	15.0	0.442
s13	ME-MP2RAGE	ME-MP2RAGE	−0.00350	12.4	8.55	8.96	0.800
s15	ME-MP2RAGE	ME-MP2RAGE	−0.000692	17.8	11.9	13.3	0.617
s18	ME-MP2RAGE	ME-MP2RAGE	0.0157	9.91	6.95	7.08	0.867
μ_g	ME-MP2RAGE	ME-FLASH	−0.142	17.9	12.9	12.5	0.581
σ_g	ME-MP2RAGE	ME-FLASH	0.139	3.44	2.59	2.28	0.140
μ_g	ME-FLASH	ME-FLASH	0.141	16.3	11.8	11.3	0.654
σ_g	ME-FLASH	ME-FLASH	0.175	2.18	1.68	1.41	0.086
μ_g	ME-MP2RAGE	ME-MP2RAGE	−0.0598	17.1	12.0	12.2	0.600
σ_g	ME-MP2RAGE	ME-MP2RAGE	0.0877	4.50	3.29	3.11	0.187
