# Supplementary material for: Simultaneous Quantitative MRI Mapping of T1, T2* and Magnetic Susceptibility with Multi-Echo MP2RAGE
Source: PLoS One. 2017 Jan 12;12(1):e0169265. doi: 10.1371/journal.pone.0169265 (PMC5230783; doi:10.1371/journal.pone.0169265)
Supplement: S11 Table — Variations of the correlation coefficients, and means and SDs of image volume differences (as defined in Eqs 4 and 5) obtained for systematic geometrical transformations of T1 maps. (PDF) [file pone.0169265.s020.pdf]

Transformation	μ_D [ms]	σ_D [ms]	$\mu_{\|D\|}$ [ms]	$\sigma_{\|D\|}$ [ms]	r^2 [#]
$R_{0.1^\circ}$	−0.00506	25.8	15.4	20.7	0.997
$R_{0.2^\circ}$	−0.00124	52.9	31.4	42.6	0.987
$R_{0.3^\circ}$	0.000909	80.9	47.9	65.2	0.969
$R_{0.4^\circ}$	−0.00390	108	64.3	87.3	0.945
$R_{0.5^\circ}$	−0.0129	134	79.9	107	0.917
$T_{0.1\text{ px}}$	−0.168	33.4	21.3	25.7	0.995
$T_{0.2\text{ px}}$	−0.317	67.1	42.8	51.6	0.979
$T_{0.3\text{ px}}$	−0.462	101	64.3	77.4	0.952
$T_{0.4\text{ px}}$	−0.613	133	85.2	102	0.917
$T_{0.5\text{ px}}$	−0.763	164	105	126	0.876
$T_{0.1\text{ px}} \circ R_{0.1^\circ}$	−0.175	42.7	26.2	33.7	0.991
$T_{0.1\text{ px}} \circ R_{0.2^\circ}$	−0.176	62.9	38.1	50.1	0.981
$T_{0.1\text{ px}} \circ R_{0.3^\circ}$	−0.172	87.5	52.7	69.9	0.964
$T_{0.1\text{ px}} \circ R_{0.4^\circ}$	−0.171	113	67.9	90.3	0.940
$T_{0.1\text{ px}} \circ R_{0.5^\circ}$	−0.177	137	82.6	109	0.913
$T_{0.2\text{ px}} \circ R_{0.1^\circ}$	−0.331	72.3	45.5	56.1	0.975
$T_{0.2\text{ px}} \circ R_{0.2^\circ}$	−0.336	85.3	52.8	67.0	0.966
$T_{0.2\text{ px}} \circ R_{0.3^\circ}$	−0.334	104	63.5	81.8	0.950
$T_{0.2\text{ px}} \circ R_{0.4^\circ}$	−0.332	124	75.9	98.5	0.928
$T_{0.2\text{ px}} \circ R_{0.5^\circ}$	−0.332	145	88.6	115	0.902
$T_{0.3\text{ px}} \circ R_{0.1^\circ}$	−0.478	104	66.2	80.3	0.949
$T_{0.3\text{ px}} \circ R_{0.2^\circ}$	−0.487	113	70.8	87.6	0.941
$T_{0.3\text{ px}} \circ R_{0.3^\circ}$	−0.490	126	78.2	98.2	0.926
$T_{0.3\text{ px}} \circ R_{0.4^\circ}$	−0.488	141	87.4	111	0.907
$T_{0.3\text{ px}} \circ R_{0.5^\circ}$	−0.487	158	97.5	124	0.885
$T_{0.4\text{ px}} \circ R_{0.1^\circ}$	−0.624	135	86.5	104	0.914
$T_{0.4\text{ px}} \circ R_{0.2^\circ}$	−0.641	141	89.5	109	0.907
$T_{0.4\text{ px}} \circ R_{0.3^\circ}$	−0.647	150	94.4	117	0.895
$T_{0.4\text{ px}} \circ R_{0.4^\circ}$	−0.648	161	101	126	0.879
$T_{0.4\text{ px}} \circ R_{0.5^\circ}$	−0.638	174	109	136	0.860
$T_{0.5\text{ px}} \circ R_{0.1^\circ}$	−0.775	165	106	127	0.874
$T_{0.5\text{ px}} \circ R_{0.2^\circ}$	−0.790	169	108	130	0.868
$T_{0.5\text{ px}} \circ R_{0.3^\circ}$	−0.800	175	111	135	0.859
$T_{0.5\text{ px}} \circ R_{0.4^\circ}$	−0.795	183	116	142	0.846
$T_{0.5\text{ px}} \circ R_{0.5^\circ}$	−0.778	193	121	150	0.830
