# Supplementary material for: Simultaneous Quantitative MRI Mapping of T1, T2* and Magnetic Susceptibility with Multi-Echo MP2RAGE
Source: PLoS One. 2017 Jan 12;12(1):e0169265. doi: 10.1371/journal.pone.0169265 (PMC5230783; doi:10.1371/journal.pone.0169265)
Supplement: S12 Table — Variations of the correlation coefficients, and means and SDs of image volume differences (as defined in Eqs 4 and 5) obtained for systematic geometrical transformations of T2* maps. (PDF) [file pone.0169265.s021.pdf]

Transformation	μ_D [ms]	σ_D [ms]	$\mu_{\|D\|}$ [ms]	$\sigma_{\|D\|}$ [ms]	r^2 [#]
$R_{0.1^\circ}$	−0.0102	1.87	0.721	1.73	0.968
$R_{0.2^\circ}$	−0.0151	2.94	1.37	2.60	0.921
$R_{0.3^\circ}$	−0.0201	3.88	1.99	3.33	0.864
$R_{0.4^\circ}$	−0.0269	4.75	2.58	3.99	0.799
$R_{0.5^\circ}$	−0.0302	5.52	3.12	4.55	0.733
$T_{0.1\text{ px}}$	−0.0269	2.70	1.12	2.45	0.934
$T_{0.2\text{ px}}$	−0.0311	4.11	2.05	3.57	0.847
$T_{0.3\text{ px}}$	−0.0230	5.26	2.90	4.40	0.754
$T_{0.4\text{ px}}$	−0.0228	6.29	3.66	5.11	0.658
$T_{0.5\text{ px}}$	−0.0346	7.17	4.35	5.70	0.569
$T_{0.1\text{ px}} \circ R_{0.1^\circ}$	−0.0236	2.95	1.29	2.65	0.921
$T_{0.1\text{ px}} \circ R_{0.2^\circ}$	−0.0220	3.59	1.73	3.15	0.883
$T_{0.1\text{ px}} \circ R_{0.3^\circ}$	−0.0244	4.33	2.25	3.70	0.831
$T_{0.1\text{ px}} \circ R_{0.4^\circ}$	−0.0294	5.07	2.77	4.24	0.772
$T_{0.1\text{ px}} \circ R_{0.5^\circ}$	−0.0312	5.76	3.27	4.75	0.710
$T_{0.2\text{ px}} \circ R_{0.1^\circ}$	−0.0241	4.23	2.14	3.65	0.839
$T_{0.2\text{ px}} \circ R_{0.2^\circ}$	−0.0164	4.59	2.39	3.91	0.812
$T_{0.2\text{ px}} \circ R_{0.3^\circ}$	−0.0163	5.09	2.74	4.29	0.770
$T_{0.2\text{ px}} \circ R_{0.4^\circ}$	−0.0200	5.65	3.15	4.69	0.720
$T_{0.2\text{ px}} \circ R_{0.5^\circ}$	−0.0202	6.22	3.56	5.10	0.667
$T_{0.3\text{ px}} \circ R_{0.1^\circ}$	−0.0215	5.34	2.95	4.45	0.747
$T_{0.3\text{ px}} \circ R_{0.2^\circ}$	−0.0136	5.57	3.11	4.62	0.727
$T_{0.3\text{ px}} \circ R_{0.3^\circ}$	−0.00631	5.91	3.34	4.88	0.695
$T_{0.3\text{ px}} \circ R_{0.4^\circ}$	−0.00411	6.32	3.63	5.18	0.656
$T_{0.3\text{ px}} \circ R_{0.5^\circ}$	−0.00520	6.75	3.94	5.48	0.613
$T_{0.4\text{ px}} \circ R_{0.1^\circ}$	−0.0218	6.34	3.70	5.14	0.653
$T_{0.4\text{ px}} \circ R_{0.2^\circ}$	−0.0137	6.48	3.80	5.25	0.639
$T_{0.4\text{ px}} \circ R_{0.3^\circ}$	−0.00507	6.71	3.95	5.43	0.616
$T_{0.4\text{ px}} \circ R_{0.4^\circ}$	−0.00321	6.99	4.14	5.64	0.588
$T_{0.4\text{ px}} \circ R_{0.5^\circ}$	−0.00232	7.30	4.36	5.86	0.556
$T_{0.5\text{ px}} \circ R_{0.1^\circ}$	−0.0345	7.20	4.37	5.73	0.565
$T_{0.5\text{ px}} \circ R_{0.2^\circ}$	−0.0260	7.30	4.43	5.80	0.555
$T_{0.5\text{ px}} \circ R_{0.3^\circ}$	−0.0168	7.45	4.53	5.92	0.540
$T_{0.5\text{ px}} \circ R_{0.4^\circ}$	−0.0130	7.64	4.65	6.06	0.520
$T_{0.5\text{ px}} \circ R_{0.5^\circ}$	−0.0117	7.85	4.80	6.22	0.498
