# Supplementary material for: Simultaneous Quantitative MRI Mapping of T1, T2* and Magnetic Susceptibility with Multi-Echo MP2RAGE
Source: PLoS One. 2017 Jan 12;12(1):e0169265. doi: 10.1371/journal.pone.0169265 (PMC5230783; doi:10.1371/journal.pone.0169265)
Supplement: S13 Table — Variations of the correlation coefficients, and means and SDs of image volume differences (as defined in Eqs 4 and 5) obtained for systematic geometrical transformations of χ maps. (PDF) [file pone.0169265.s022.pdf]

Transformation	μ_D [ppb]	σ_D [ppb]	$\mu_{\|D\|}$ [ppb]	$\sigma_{\|D\|}$ [ppb]	r^2 [#]
$R_{0.1^\circ}$	0.00227	2.20	1.37	1.72	0.993
$R_{0.2^\circ}$	0.00653	4.49	2.80	3.51	0.972
$R_{0.3^\circ}$	0.0110	6.83	4.26	5.34	0.936
$R_{0.4^\circ}$	0.0136	9.07	5.69	7.06	0.887
$R_{0.5^\circ}$	0.0141	11.1	7.04	8.58	0.833
$T_{0.1\ \mathrm{px}}$	0.00548	3.35	2.24	2.49	0.984
$T_{0.2\ \mathrm{px}}$	0.0175	6.68	4.49	4.95	0.938
$T_{0.3\ \mathrm{px}}$	0.0311	9.96	6.72	7.35	0.863
$T_{0.4\ \mathrm{px}}$	0.0432	13.1	8.87	9.62	0.768
$T_{0.5\ \mathrm{px}}$	0.0484	16.0	10.9	11.7	0.664
$T_{0.1\ \mathrm{px}} \circ R_{0.1^\circ}$	0.00808	4.19	2.68	3.22	0.976
$T_{0.1\ \mathrm{px}} \circ R_{0.2^\circ}$	0.0119	5.82	3.66	4.52	0.953
$T_{0.1\ \mathrm{px}} \circ R_{0.3^\circ}$	0.0160	7.79	4.89	6.06	0.916
$T_{0.1\ \mathrm{px}} \circ R_{0.4^\circ}$	0.0188	9.80	6.18	7.60	0.869
$T_{0.1\ \mathrm{px}} \circ R_{0.5^\circ}$	0.0212	11.7	7.43	9.01	0.816
$T_{0.2\ \mathrm{px}} \circ R_{0.1^\circ}$	0.0194	7.21	4.76	5.41	0.928
$T_{0.2\ \mathrm{px}} \circ R_{0.2^\circ}$	0.0230	8.25	5.35	6.28	0.906
$T_{0.2\ \mathrm{px}} \circ R_{0.3^\circ}$	0.0270	9.65	6.20	7.40	0.872
$T_{0.2\ \mathrm{px}} \circ R_{0.4^\circ}$	0.0306	11.2	7.20	8.60	0.829
$T_{0.2\ \mathrm{px}} \circ R_{0.5^\circ}$	0.0334	12.8	8.22	9.75	0.781
$T_{0.3\ \mathrm{px}} \circ R_{0.1^\circ}$	0.0340	10.3	6.91	7.67	0.853
$T_{0.3\ \mathrm{px}} \circ R_{0.2^\circ}$	0.0377	11.0	7.30	8.26	0.834
$T_{0.3\ \mathrm{px}} \circ R_{0.3^\circ}$	0.0407	12.0	7.86	9.02	0.805
$T_{0.3\ \mathrm{px}} \circ R_{0.4^\circ}$	0.0452	13.1	8.57	9.90	0.769
$T_{0.3\ \mathrm{px}} \circ R_{0.5^\circ}$	0.0471	14.3	9.34	10.8	0.729
$T_{0.4\ \mathrm{px}} \circ R_{0.1^\circ}$	0.0461	13.3	9.02	9.84	0.760
$T_{0.4\ \mathrm{px}} \circ R_{0.2^\circ}$	0.0493	13.8	9.27	10.2	0.744
$T_{0.4\ \mathrm{px}} \circ R_{0.3^\circ}$	0.0526	14.4	9.64	10.7	0.722
$T_{0.4\ \mathrm{px}} \circ R_{0.4^\circ}$	0.0565	15.2	10.1	11.3	0.694
$T_{0.4\ \mathrm{px}} \circ R_{0.5^\circ}$	0.0583	16.0	10.7	12.0	0.663
$T_{0.5\ \mathrm{px}} \circ R_{0.1^\circ}$	0.0514	16.2	11.0	11.9	0.657
$T_{0.5\ \mathrm{px}} \circ R_{0.2^\circ}$	0.0565	16.5	11.2	12.1	0.646
$T_{0.5\ \mathrm{px}} \circ R_{0.3^\circ}$	0.0609	16.9	11.4	12.4	0.630
$T_{0.5\ \mathrm{px}} \circ R_{0.4^\circ}$	0.0632	17.4	11.7	12.8	0.610
$T_{0.5\ \mathrm{px}} \circ R_{0.5^\circ}$	0.0640	17.9	12.1	13.3	0.588
