# Supplementary material for: Simultaneous Quantitative MRI Mapping of T1, T2* and Magnetic Susceptibility with Multi-Echo MP2RAGE
Source: PLoS One. 2017 Jan 12;12(1):e0169265. doi: 10.1371/journal.pone.0169265 (PMC5230783; doi:10.1371/journal.pone.0169265)
Supplement: S14 Table — Variations of the correlation coefficients, and means and SDs of image volume differences (as defined in Eqs 4 and 5) obtained for systematic increase of the noise level for the complex images used to compute χ maps. (PDF) [file pone.0169265.s023.pdf]

Noise level [arb.unit]	μ_D [ppb]	σ_D [ppb]	$\mu_{\|D\|}$ [ppb]	$\sigma_{\|D\|}$ [ppb]	r^2 [#]
1	0.000131	0.593	0.452	0.384	1.000
5	0.000628	2.92	2.25	1.87	0.988
10	−0.000963	5.79	4.48	3.69	0.955
15	0.00277	8.63	6.69	5.47	0.903
20	0.00728	11.4	8.86	7.22	0.838
25	0.0124	14.2	11.0	8.93	0.764
30	0.0167	16.8	13.1	10.6	0.688
